# 16S rDNA Pyrosequencing Analysis of Bacterial Community in Heavy Metals Polluted Soils

**DOI:** 10.1007/s00248-013-0344-7

**Published:** 2014-01-09

**Authors:** Marcin Gołębiewski, Edyta Deja-Sikora, Marcin Cichosz, Andrzej Tretyn, Borys Wróbel

**Affiliations:** 1 Chair of Plant Physiology and Biotechnology, Nicolaus Copernicus University, Lwowska 1, 87-100 Toruń, Poland; 2 Centre of Modern Interdisciplinary Technologies, Nicolaus Copernicus University, Wilenska 4, 87-100 Toruń, Poland; 3 Institute of Oceanology PAS, Powstańców Warszawy 55, 81-712 Sopot, Poland; 4 Evolutionary Systems Laboratory, Adam Mickiewicz University, Umultowska 89, Room S1, 61-614 Poznań, Poland; 5 Department of Chemical Pro-ecological Processes, Nicolaus Copernicus University, Gagarina 7, 87-100 Toruń, Poland

## Abstract

Soil contamination with heavy metals is a widespread problem, especially prominent on grounds lying in the vicinity of mines, smelters, and other industrial facilities. Many such areas are located in Southern Poland; they are polluted mainly with Pb, Zn, Cd, or Cu, and locally also with Cr. As for now, little is known about most bacterial species thriving in such soils and even less about a core bacterial community—a set of taxa common to polluted soils. Therefore, we wanted to answer the question if such a set could be found in samples differing physicochemically and phytosociologically. To answer the question, we analyzed bacterial communities in three soil samples contaminated with Pb and Zn and two contaminated with Cr and lower levels of Pb and Zn. The communities were assessed with 16S rRNA gene fragments pyrosequencing. It was found that the samples differed significantly and Zn decreased both diversity and species richness at species and family levels, while plant species richness did not correlate with bacterial diversity. In spite of the differences between the samples, they shared many operational taxonomic units (OTUs) and it was possible to delineate the core microbiome of our sample set. The core set of OTUs comprised members of such taxa as *Sphingomonas*, *Candidatus Solibacter*, or *Flexibacter* showing that particular genera might be shared among sites ~40 km distant.

**Electronic supplementary material:**

The online version of this article (doi:10.1007/s00248-013-0344-7) contains supplementary material, which is available to authorized users.

## Introduction

Analysis of biodiversity in areas under anthropogenic pressure (e.g., pollution or climate change) allows to quantify the possibly detrimental effects of human activity on particular taxonomic groups. Due to the rapid response, microbial biodiversity may also be used to monitor the outcome of bioremediation/bioreclamation efforts [1].

A number of existing studies showed varying degree of heavy metals impact on soil bacteria. Frostegård and colleagues [2] found that Cd, Cu, Ni, Pb, and Zn influenced patterns of phospholipid fatty acids (PLFA) and decreased biomass and activity (measured as respiration) of microbial communities in forest and arable soils. However, determination which bacterial species or higher taxonomic units were affected was beyond the capabilities of this methodology. rRNA-based methods allowed for more detailed analyses; certain groups reported differential influence of metals on culturable and unculturable fractions of soil bacterial community [3, 4]. Total community richness was found to be only moderately affected by heavy metals [5, 6], but Gans et al. found that the diversity in heavily polluted soils may constitute only 1 % of that in pristine soils [7].

It is estimated that only 0.1–1 % of soil bacteria are readily culturable [8, 9]. This leads to the conclusion that culture-independent methods should be preferred, although some researchers disagree with this view claiming that the culturable subcommunity is the most active, and therefore the most important [4, 10, 11].

Among the methods that can assess both culturable and unculturable bacteria, the majority is based on marker genes, such as 16S rRNA or *recA* genes [12, 13]. Next generation sequencing (NGS) quickly becomes the method of choice, due to the high data yield and relatively low costs of data generation. To date, several reports on soil bacterial communities analyzed via 16S rRNA gene fragments pyrosequencing were published [14–17], but only a few of them were devoted to metals-contaminated soils. The dependence of bacterial diversity and species richness on the contamination was found in Cd, Pb, and Zn [18, 19] as well as Cr and As [20] and Cu [19] contaminated soils. Metals caused decrease of diversity, increase of Proteobacteria, and decrease of Acidobacteria and Actinobacteria levels. However, when considered with other factors, they were not the most important variables shaping the community structure [19].

We wanted to see if bacterial communities in two soils, differently contaminated with heavy metals and varying in other physicochemical parameters as well as phytosociologically but coming from the same geographical region were similar. Basing on the shared operational taxonomic units (OTUs) set, we delineated a core microbiome of our samples, defined as the set of OTUs evenly distributed among all the samples and present at the percentage greater than 0.1 %.We looked also at the diversity, the species richness, and the community structure. To study the parameters of interest, barcoded libraries of V3–V4 16S rRNA gene fragments were pyrosequenced and between 15,000 and 20,000 raw reads from each sample were generated. We developed a bioinformatic pipeline based on MOTHUR [21] and used it to analyze the parameters mentioned above.

## Methods

### Soil Samples

Samples were collected at two sites in Southern Poland: near Olkusz (50°17′ N, 19°30′ E), in the vicinity of a lead and zinc ore enrichment facility (specifically its flotation waste collector), and in Alwernia (50°02′ N, 19°31′ E), near a chromium green-producing facility. The sampling points were located on public grounds, not protected by nature reserve or national park, and therefore no specific permission was required. The fieldwork did not involve any endangered or protected species.

Phytosociological analysis of the sampling sites was performed according to the Braun-Blanquet method [22]. Briefly, one phytosociological picture was taken at each site, then the plant species were identified and their coverage (in standard six-point Braun-Blanquet scale: +—rare species, 1—abundant species, coverage lower than 20 %, 2—very abundant species with coverage <20 % or abundant one with coverage between 20 and 25 %, 3—25–50 % coverage, 4—50–75 % coverage, 5—75–100 % coverage) was estimated.

Top soil from the 5–30-cm layer was collected in July 2009. Five subsamples were taken at each site at the four vertices and in the center of a 2.5-m square. After visible plant fragments and large stones were removed, the subsamples were combined, and the sample was thoroughly mixed with a spade. After sampling, the soils were transferred to plastic bags which were sealed and stored in a cold room (at 9 ºC) until analysis. DNA isolation (details below) was performed within 5 days, other analyzes until the end of 2009. The soil texture was assessed with the laser diffraction method on Analysette 22 (Fritsch, Germany). The organic matter and carbonates content were estimated by loss on ignition at 550 and 950 ºC, respectively. The pH was measured in a 1:10 soil-water slurry with a digital pH meter (Elmetron, Poland). The metals content was analyzed by X-ray fluorescence (XRF), and their bioavailable concentrations were determined with atomic absorption spectrometry (AAS) after DETAPAC extraction [23]. The samples characteristics are summarized in Table 1, and the phytosociological analysis can be found in Table 2.

**Table 1:** Soil samples characteristics

Name	GPS coordinates	Soil type	OM (%)	C_ino_^a^ (%)	P_2_O_5_ (%)	H_2_O (%)	pH	Fe_2_O_3_ (%)	Cd_t_^b^ (mg/kg)	Cd_ba_^c^ (mg/kg)	Pb_t_ (mg/kg)	Pb_ba_ (mg/kg)	Zn_t_ (mg/kg)	Zn_ba_ (mg/kg)	Cr_t_ (mg/kg)	Cr_ba_ (mg/kg)
A1	50º 02′ 54,8″ N, 19º 31′ 34,2″ E	Sand	2.82	0.12	0.02	2.18	7.0	2.23	4.2 ± 0.2	2.5 ± 0.1	204 ± 5	104 ± 5	749 ± 5	521 ± 5	740 ± 10	492 ± 10
A2	50º 02′ 40,4″ N, 19º 31′ 38,4″ E	Silt	4.62	0.09	0.09	2.77	6.5	3.23	3.5 ± 0.2	2.1 ± 0.1	203 ± 5	99 ± 5	767 ± 5	538 ± 5	760 ± 10	501 ± 10
O1	50º 17′ 21,0″ N, 19º 30′ 48,1″ E	Sand	19.90	0.30	0.13	5.60	6.7	2.02	3.4 ± 0.2	2.1 ± 0.1	1,378 ± 5	730 ± 5	1,247 ± 5	863 ± 5	100 ± 10	63 ± 10
O2	50º 17′ 17,2″ N, 19º 30′ 28,8″ E	Sand	16.23	0.62	0.05	2.95	6.5	1.06	3.9 ± 0.2	2.3 ± 0.1	461 ± 5	241 ± 5	957 ± 5	692 ± 5	180 ± 10	121 ± 10
O3	50º 17′ 15,2″ N, 19º 30′ 11,7″ E	Sand	3.82	30.37	0.01	1.07	7.6	7.43	3.7 ± 0.2	2.2 ± 0.1	1,097 ± 5	563 ± 5	2,002 ± 5	1,478 ± 5	300 ± 10	196 ± 10

^a^Inorganic carbon content

^b^Total concentration

^c^Bioavailable concentration

**Table 2:** Phytosociological analysis of sampling points

Sample	Phytosociological classification	Plant sp. richness	Trees coverage	Herbaceous plants coverage	Main species
A1	No-rank *Equisetum arvense* Class: *Agropyretea intermedio-repentis*	12	0 %	15 % + 10 % mosses	*Equisetum arvense*
A2	Assoc.: *Phleum pratense* Alliance: *Cynosurion* Order: *Arrhenatheretalia* Class: *Molinio-Arrhenatheretea*	36	0 %	100 %	*Phleum pratense*
O1	Assoc.: *Querco roboris-Pinetum* Alliance: *Dicrano-Pinion* Order: *Piceetalia abietis* Class: *Vaccinio-Piceetaea*	35	65 %	80 %	*Pinus sylvestris, Festuca ovina, Leontodon hispidus, Rumex thyrsiflorus*
O2	Assoc.: *Spergulo vernalis-Corynephoretum* Alliance: *Corynephorion canescentis* Order: *Corynephoretalia canescentis* Class: *Koelerio-Corynephoretea*	14	5 % (shrubs)	20 %	*Pinus sylvestris, Corynephorus canescens*
O3	No-rank *Gypsophila fastigiata* Class: *Koelerio-Corynephoretea*	14	0 %	15 %	*Gypsophila fastigiata*

### Analysis of 357f and 786r Primers Coverage and Specificity

Primers’ coverage (percentage of bacterial sequences in a database from which the primer set generates amplicons) and specificity (percentage of nonbacterial sequences amplified by the primers) were assessed by TestProbe from the SILVA website (http://beta.arb-silva.de/search/testprobe; [24]) run on the SSU r114 database and by Ribosomal Database Project’s tool ProbeMatch (http://rdp.cme.msu.edu/probematch/search.jsp; [25]) run against the RDP 10 update 31 database. Both tools were run with two mismatches allowed and otherwise default settings, with the exception of using weighted mismatches in TestProbe. The parameters for the pair of primers were also assessed with ProbeMatch of RDP and TestPrime from SILVA [26].

### Isolation of Metagenomic DNA

Metagenomic DNA was isolated from 30 g of homogenized soils with the method developed by Zhou and colleagues [27] modified to increase the yield of DNA and the spectrum of lysed bacteria. The modifications involved adding an enzymatic digestion (lysozyme and achromopeptidase) step before the SDS lysis and lowering the lysis temperature (55 ºC instead of 65 ºC) [28]. Subsequently, the DNA was purified in two agarose gel electrophoresis steps, first using 0.7 %, and then in 1 % agarose. Pure DNA was recovered from the gel with an agarose gel extraction kit (Roche). The quality of the preparations was assessed spectrophotometrically on NanoDrop ND-1000 (NanoDrop, Wilmington, DE).

### PCR Amplification of 16S rRNA Gene Fragments and Pyrosequencing

A fragment of the 16S rRNA gene encompassing the V3 and V4 variable regions [29] was amplified with primers 357f (5′ CCT ACG GGA GGC AGC AG 3′, [30]) and 786r (5′ AC CAG GGT ATC TAA WCC 3′, this work) with overhangs required for pyrosequencing. The forward primer was used in three versions differing in the multiplex identifier (MID) sequence between the A adaptor and the actual primer. The reaction mix contained the following: 1 ng of template DNA, 10 pmol primers, 0.2 mM dNTP, 1.5 mM MgCl_2_, 0.2 U of Taq polymerase, and the appropriate 1× buffer (Fermentas, Lithuania). The cycling conditions were as follows: 95 ºC for 5 min, 30 cycles of: 95 ºC for 30 s, 52 ºC for 30 s, 72 ºC for 30 s; and finally 72 ºC for 2 min. The reactions were carried out in MasterCycler thermocycler (Eppendorf, Germany). Eight independent reactions were performed for each sample.

PCR products were purified with the DNA CleanUp kit (A&A Biotechnology, Poland), pooled in equimolar amounts, vacuum dried, and either sent for sequencing at Max Planck Molecular Genetics Institute sequencing service (Berlin, Germany—Olkusz samples) or sequenced at the Chair of Genetics, University of Silesia (Katowice, Poland—Alwernia ones). Pyrosequencing was performed with the use of Titanium chemistry on GS FLX and GS Junior instruments (Roche, USA) as described in the manufacturer’s protocols.

### Bioinformatic Analysis

We developed a bash script that allowed a streamlined analysis of sequence data using MOTHUR v.1.30.2 [21] and R [31] and by calling other scripts in bash. The script took as the input an .sff file(s) (either a single one with all samples or many single-sample files) and a file containing information on the primers used for amplicons generation with optional barcodes (in the case of single .sff) or, in the case of multiple .sff files, additional file with sample names. The computations were parallelized where possible to speed up the analysis. The limits on the number of processors and the amount of RAM to be used in the clustering step could be set to allow running the script on multi-processors machines without consuming all the resources available.

The script can be run on any POSIX-compatible operating system, where installation of bash shell is possible. We ran the analyzes on a 48-core 256 GB RAM machine under control of the 64-bit version of Fedora 14 Linux, using eight cores and with limit of RAM amount set to 10 GB. All the procedures described below were pipelined in this script, available from the authors on request. The actual parameters for all steps of the pipeline can be found in Supplementary Materials.

Pyrosequencing adaptor and MID sequences were removed from the Olkusz reads by the sequencing center, and quality trimming was performed for separate sample files. For the Alwernia samples, a single .sff file was obtained, and the trimming was done accordingly. Quality trimming was performed with the AmpliconNoise algorithm [32] implemented in MOTHUR’s shhh.flows. The reads were then dereplicated, aligned against the SILVA template alignment, and screened for those covering the desired alignment region. The gap-only and terminal gap-containing columns were filtered out of the alignment and putative chimeras were identified with the uchime algorithm [33] and removed. Finally, the sequencing noise was further reduced via single-linkage pre-clustering [34]. Then, the high-quality reads were down-sampled to the smallest sample size and (1) classified with naïve Bayesian classifier implemented in MOTHUR (classify.seqs) using SILVA taxonomy provided by the authors of the program on the webpage www.mothur.org/w/images/9/98/Silva.bacteria.zip, as well as (2) a distance matrix was calculated, and OTUs were constructed by average neighbor clustering. Sequences were regarded successfully classified at a given level if their bootstrap support was greater than 80 %.

### Species Richness and Diversity Analyzes

Species richness was assessed with Chao1 [35] and ACE [36] indices, as well as with catchall v3.0 [37]. Phylogenetic diversity was measured according to the methodology of Faith and coworkers [38] (MOTHUR’s command “phylo.diversity”). The estimators were calculated for five subsamples containing 1,500 sequences drawn randomly from each group and averaged to yield the final values.

An averaged shared OTU table was prepared from the subsamples (for details, see Supplementary Materials).

Morisita-Horn [39] and Bray-Curtis [40] dissimilarities were calculated for all the pairs of samples via dist.shared command of MOTHUR. They were visualized as heatmaps with the use of the heatmap.sim command. Venn diagrams were prepared from the shared OTU table using a custom Perl script.

To check if the communities differed significantly, the parsimony [41] and weighted UniFrac [42] tests were employed. The MOTHUR commands parsimony and unifrac.weighted were used, respectively. The tests were run on a phylogenetic tree generated from the same distance matrix that served for the OTU construction, with the use of the relaxed neighbor joining algorithm of clearcut [43] implemented in MOTHUR.

### Statistical Analysis

Statistical analysis was performed in R 2.15.2 [31], using vegan [44] (redundancy analysis (RDA) with significance testing by permutation—ANOVA) and Hmisc [45] packages (Spearman’s correlation in rcorr).

### Core Microbiome Delineation

The “core microbiome,” as defined by Turbaugh and coworkers [46], would consist of the OTUs that were present in all the samples. We added two conditions here: (1) the OTUs should be present in roughly equal amounts and (2) the frequency of reads has to be >0.1 % in each sample (>2 reads). OTUs meeting the conditions were identified by calculation of Shannon’s evenness, treating the samples as taxa and reads as individuals and eliminating those with two or less reads in any sample. The OTUs with the value greater than 0.9 were regarded “evenly distributed” and assigned to the core microbiome.

## Results

### Sites and Soils Characteristics

In this study, we analyzed the bacterial community structure and species richness in three soil samples from Olkusz and two from Alwernia, both towns located in the Malopolska province of Poland. The soils came from the area lying in the vicinity of a flotation waste pond of a zinc and lead ore enrichment facility (Olkusz) and from the grounds adjacent to a factory producing chromium-based pigments (Alwernia).

The samples differed from each other, both in terms of plant species richness (A1 being the least rich, and A2 being the most) and phytosociological classification (Table 2). Also, the ground coverage by plants varied greatly, from almost 0 % for O3 to 100 % for A2. Two samples were classified as no-rank associations (A1—no rank *Equisetum arvense*, O3—no rank *Gypsophila fastigiata*), while the remaining three belonged to syntaxons well placed in the hierarchy (Table 2).

The three Olkusz samples appeared to be sandy (Table 1), the organic matter content varied between 19.90 % (O1) and 3.82 % (O3), and the inorganic carbon (carbonates) content was below 1 % in O2 and O3, while it was over 30 % in O3. High C_org_ content in the O1 and O2 samples was due to the addition of peat to the upper layer of soil in an effort to immobilize heavy metal ions to allow land reclamation, which was done in 1990s [47]. High level of carbonates in O3 was brought about by dolomite, which was the main component of flotation waste. The pH was close to neutral in all samples. Slightly alkaline pH in the O3 sample might have resulted from the high dolomite content and the remnants of the flotation reagents (xanthates) leaking from the pond. The organic matter content was 2.82 and 4.62 % in A1 and A2, respectively, the carbonates were low, and the pH was close to neutral.

The samples were only moderately contaminated with Cd (3.4–4.2 mg kg^−1^ total conc., 2.1–2.5 mg kg^−1^ bioavailable conc.—Table 1), and the sites did not differ much in the concentration of this metal. The levels of Pb and, particularly, Zn were high in the Olkusz samples, reaching 1,097 mg kg^−1^ (Pb) and 2,002 mg kg^−1^ (Zn) total concentration at O3, and were much lower in Alwernia ones. The opposite pattern was found for Cr, whose concentration was highest in A2 (760 mg kg^−1^). Bioavailable concentrations of the metals were at the level of 70 % of the total concentrations. It seems that in the Olkusz samples, Pb and Zn were the main contaminants which might have influenced bacterial communities. In the Alwernia ones, Cr and Zn were the dominant pollutants, while Cd could be neglected in all the samples due to its low concentration.

### Primers’ Coverage and Specificity

The 357f primer was highly specific, as only 2.5 % of matches came from Archaea according to RDP and 20.6 % were not from bacteria according to SILVA. It had also high coverage, as it amplified over 99.4 % (RDP) and 96.7 % (SILVA) of bacterial sequences. The 786r primer also performed well, having 99.3 and 97.2 % of coverage among bacteria (according to RDP and SILVA, respectively) and slightly lower specificity (5.4 and 26.1 % of matches were nonbacterial in RDP and SILVA). Both tools reported a fair coverage of Archaea by the primers (up to 86.6 % for Euryarchaeota), but when no mismatches were allowed, these matches disappeared.

As a pair, the primers were highly specific for bacteria (2.9 and 25.7 % nonbacterial matches in RDP and SILVA, respectively) and the coverage was also high (98.8 and 88.7 %). The values reported were calculated excluding sequences lacking the 357–786 region.

### Sequencing

The DNA preparations were of high quality, as their A260/280 ratios were ~1.8 for all samples and A260/230 at ~2 indicating high purity. All of them performed well as PCR templates. In total, 66,385 reads were obtained (Table 3), out of which 25,808 (38.9 %) met the quality criteria. The 2, 376 potential chimeras were removed, and 23,432 (35.3 % of the total number) high-quality nonchimeric reads were included in the downstream analysis. The median of read length was 250 bp and the mean was 255 bp. All the reads were successfully classified at the domain (all of them came from bacteria) and phylum level (bootstrap support >80 %), for lower taxonomic levels, the percentage of unclassified sequences increased, to reach 50.5 % for genus. The reads generated in this project were deposited in the NCBI’s SRA database under SRA IDs SRA024301.1 (Olkusz) and SRA059341.1 (Alwernia).

**Table 3:** Sequencing statistics

Sample	Raw reads no.	High-quality reads		Chimeras		High-quality non-chimeric reads	
		Number	%^a^	Number	%^b^	Number	%^a^
A1	4,235	3,054	72.1 %	422	13.8 %	2,632	62.1 %
A2	5,947	4,229	71.1 %	783	18.5 %	3,446	57.9 %
O1	15,967	7,474	46.8 %	412	5.5 %	7,062	44.2 %
O2	17,522	6,215	35.5 %	363	5.8 %	5,852	33.4 %
O3	22,714	4,836	21.2 %	396	8.2 %	4,440	19.5 %
**Total**	**66,385**	**25,808**	**38.9 %**	**2 376**	**9.2 %**	**23,432**	**35.3 %**

^a^Percent of raw reads

^b^Percent of high-quality reads

### Community Species Richness and Diversity Indices

**Fig. 1:**
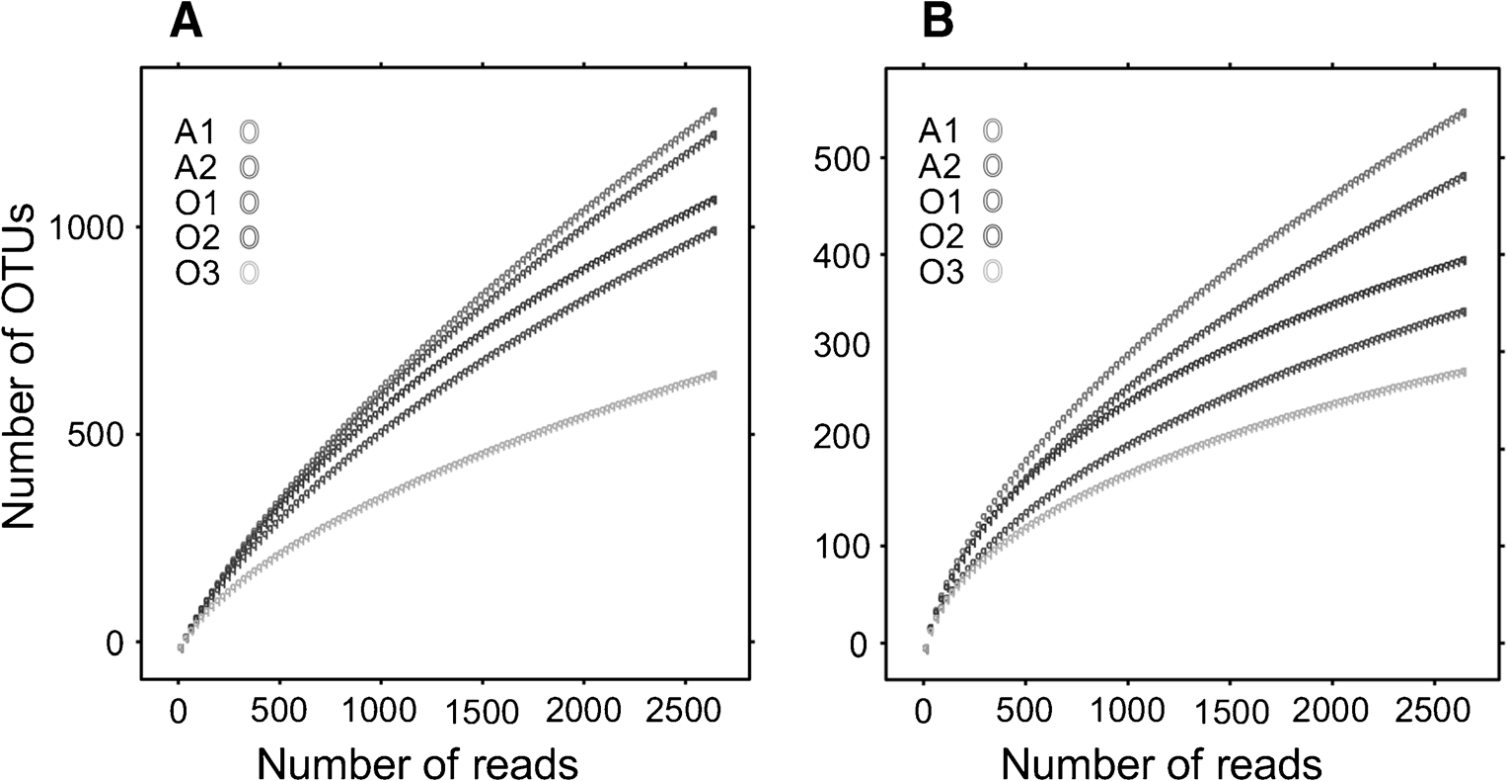
Rarefaction curves. **a** 0.03 dissimilarity level, **b** 0.10 dissimilarity level

The communities were moderately sampled, indicating that much more reads would be required to capture all the diversity (Fig. 1). The Alwernia samples were more diverse than the Olkusz ones, which was particularly visible at the 0.10 level (Table 4). Bacterial diversity in the Olkusz soils decreased with increasing concentration of Pb and Zn (O2 > O1 > O3), while A1 was more diverse than A2 in spite of the higher contamination level. The rarefaction analysis (Fig. 1) gave results similar to the comparison of Shannon’s H′ (Table 5). Evenness in all samples was close to 0.9 for the 0.03 clustering level and in the range 0.7–0.8 for the 0.10 level, the highest values were observed in A1.

**Table 4:** Species richness estimators

Sample	OTUs observed		Chao1		ACE		Catchall^b^	
	0.03^a^	0.10^a^	0.03	0.10	0.03	0.10	0.03	0.10
A1	**858**	**386**	**3,025** (2,536–3,657)	**981** (790–1,261)	**5,188** (4,747–5,679)	**1,554** (1,381–1,758)	**8,637**	1,747
A2	826	342	2,530 (2,150–3,019)	881 (697–1,160)	4,515 (4,139–4,934)	1,347 (1,195–1,527)	6,662	**1,900**
O1	699	263	1,589 (1,383–1,858)	436 (372–536)	2,596 (2,361–2,864)	576 (504–670)	2,558	610
O2	749	312	1,728 (1,507–2,013)	523 (448–640)	2,723 (2,485–2,993)	637 (569–723)	3,239	900
O3	476	224	1,069 (903–1,301)	402 (330–521)	1,768 (1,589–1,977)	571 (495–667)	1,934	546

Each value is a mean of five obtained for random subsamples of equal size, 95 % confidence intervals are in parentheses. Highest values are in boldface

^a^Dissimilarity level for OTU construction

^b^Due to a bug in catchall there is no confidence intervals for the estimations

**Table 5:** Diversity and evenness estimators

Sample	Shannon’s H′		Shannon’s evenness		Phylodiversity
	0.03^a^	0.10^a^	0.03	0.10	
A1	**6.367 (6.309–6.425)**	**4.984 (4.908–5.060)**	**0.943**	0.837	**53,34**
A2	6.309 (6.249–6.368)	4.800 (4.723–4.878)	0.939	0.823	49.14
O1	5.978 (5.909–6.047)	4.280 (4.194–4.366)	0.913	0.768	40,32
O2	6.196 (6.137–6.254)	4.810 (4.738–4.883)	0.936	**0.838**	45,63
O3	5.321 (5.247–5.395)	4.169 (4.087–4.251)	0.863	0.771	30,66

Each value is an average of five coming from random subsamples of size 1,500. 95 % confidence intervals are given in parentheses

^a^Dissimilarity level for OTU construction

Both the observed and estimated total richness was higher in the Alwernia communities than in the Olkusz ones (Table 4). At the 0.03 dissimilarity level, all the methods predicted the highest richness in A1, while at the 0.10 level, only catchall predicted a greater number of OTUs in A2 than in A1. The parametric methods, such as catchall, should be independent of the community coverage, therefore they could be trusted more than the nonparametric estimators. The collectors curves for the Chao1 and ACE indices showed no saturation (Fig. S2), so one would expect that the values of these estimators would increase with the sampling effort. In other words, they should not be treated as the true richness estimations.

The estimated number of OTUs in the least rich sample (O3) was about five times lower than in the richest one (A1 or A2). As all the methods used predicted that the richness was smallest in the most contaminated sample (O3), it might mean that species richness was affected by heavy metals contamination. Indeed, regression analysis showed that the Zn concentration negatively correlated with all estimators of species richness and diversity, and the correlation seemed to be nonlinear (Spearman’s rank correlation, *p* < 0.05, Table 6, Supplementary Fig. S1). However, due to the low number of samples, such results should be treated cautiously, in spite of their high statistical significance. No other variables, including the phosphorus concentration and the plant species richness, correlated with bacterial diversity (RDA model’s *p* > 0.05) or species richness measures (Spearman’s rank correlation, *p* > 0.05, Table S3).

**Table 6:** Correlation of zinc concentration with bacterial species richness and diversity estimators

	Spearman’s correlation coefficient and *p* value
Sp. observed (OTUs 0.03)	ρ = −1, *p* = 0
Chao1 (OTUs 0.03)	ρ = −1, *p* = 0
ACE (OTUs 0.03)	ρ = −1, *p* = 0
Shannon’s H′ (OTUs 0.03)	ρ = −1, *p* = 0
Sp. observed (OTUs 0.10)	ρ = −1, *p* = 0
Chao1 (OTUs 0.10)	ρ = −1, *p* = 0
ACE (OTUs 0.10)	ρ = −1, *p* = 0
Shannon’s H′ (OTUs 0.10)	ρ = −0.90, *p* = 0.03
Phylodiversity	ρ = −1, *p* = 0

### Bacterial Community Structure

**Fig. 2:**
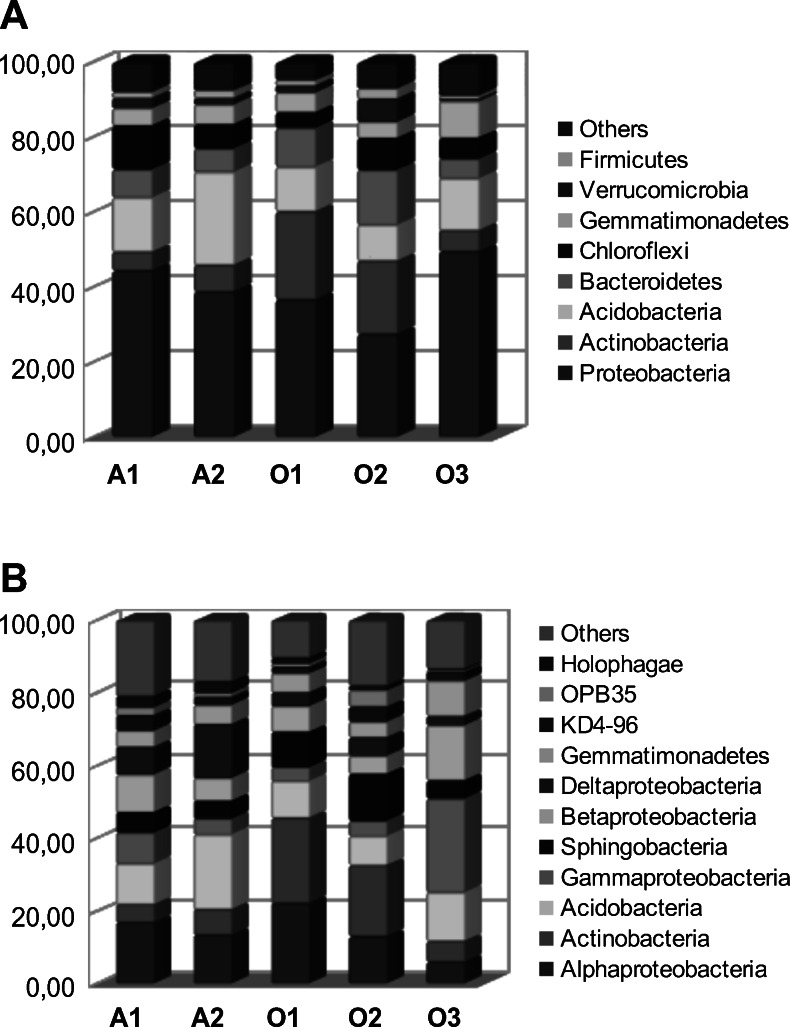
Bacterial community structure in samples. **a** Phylum level, **b** class level

**Fig. 3:**
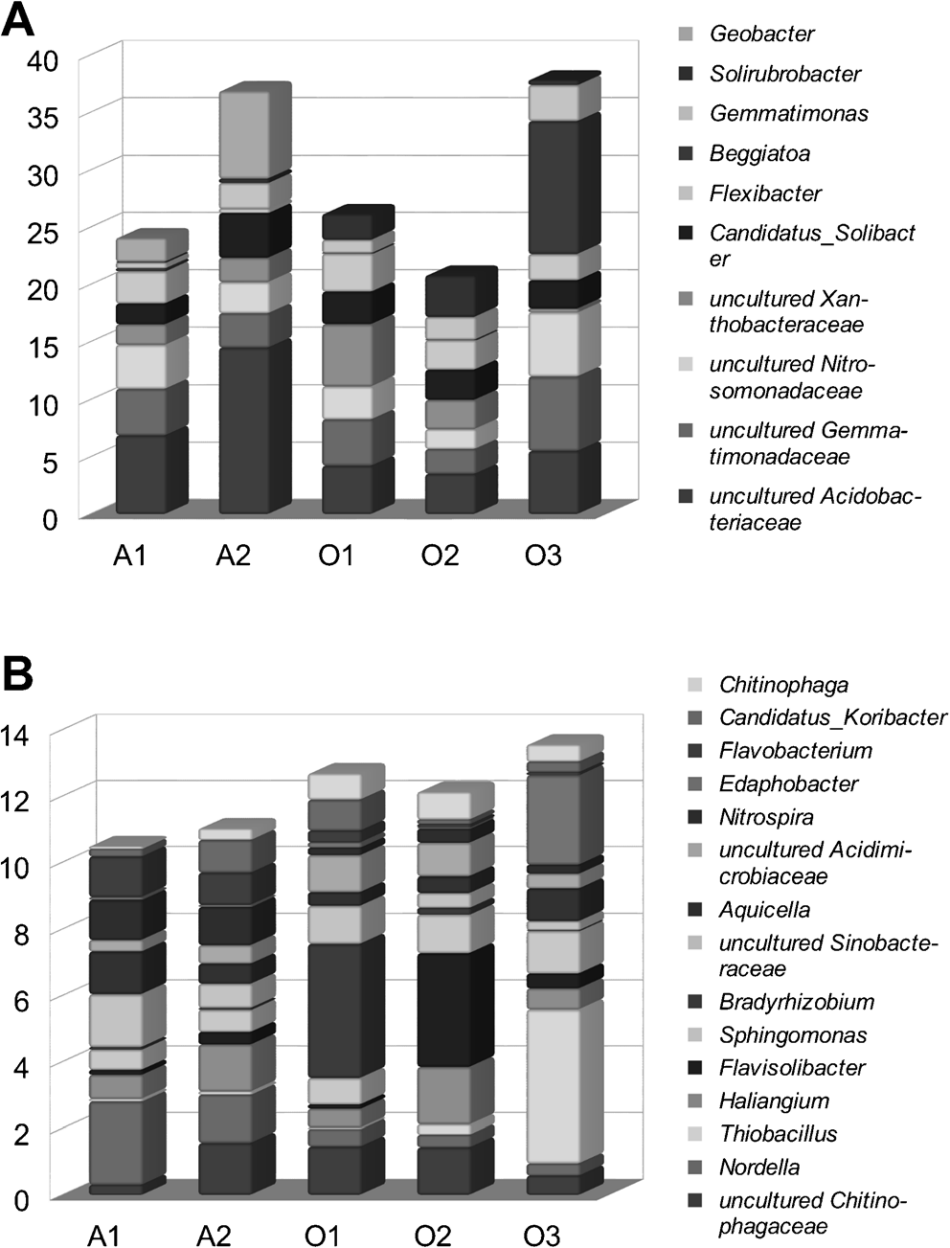
Most abundant genera in samples. **a** 10 genera of highest total abundance; **b** next 15 genera

All the reads were assigned to bacteria. Proteobacteria were the most abundant phylum in all five samples (27.5–49.5 %—Fig. 2, Table S1), in the Alwernia ones and O3, they were followed by Acidobacteria, while in O1 and O2 by Actinobacteria. Other major phyla were Bacteroidetes, Chloroflexi, and Gemmatimonadetes. The most abundant classes were Alphaproteobacteria, Actinobacteria, Acidobacteria, Gammaproteobacteria, Sphingobacteria, and Betaproteobacteria (Fig. 3b). The most abundant genera (>100 reads across all samples) collectively comprised 43.1 % of all reads, and their percentage varied from 35.8 % (O2) to 55.4 % (O3) (Fig. 3, details in Table S2). The four most abundant ones were uncultured members of Acidobacteriaceae, Gemmatimonadaceae, Nitrosomonadaceae, and Xanthobacteraceae. The Alwernia samples contained more representatives of an uncultured genus of Acidobacteriaceae family and *Geobacter* (Deltaproteobacteria, Geobacteraceae) than the Olkusz ones, while genus *Solirubrobacter* (Actinobacteria, Solirubrobacteraceae) was more abundant in the Olkusz soils. The O3 sample contained a high proportion of sulfur oxidizing bacteria of genera *Beggiatoa* (Gammaproteobacteria, Thiotrichaceae) and *Thiobacillus* (Betaproteobacteria, Hydrogenophilaceae) that probably utilized sulfides present in mine tailings, mainly pyrite, and possibly organosulfur compounds used in the flotation process (xanthates).

Influence of environmental variables on the community structure was assessed with RDA. Zn, Pb, Cr, and pH were found to be significantly correlated with the community structure both on the 0.10 and 0.03 clustering levels (*p* < 0.05, see Fig. S3). Notably, the P concentration and plant species richness did not influence the communities under study.

### Comparison of Communities

**Fig. 4:**
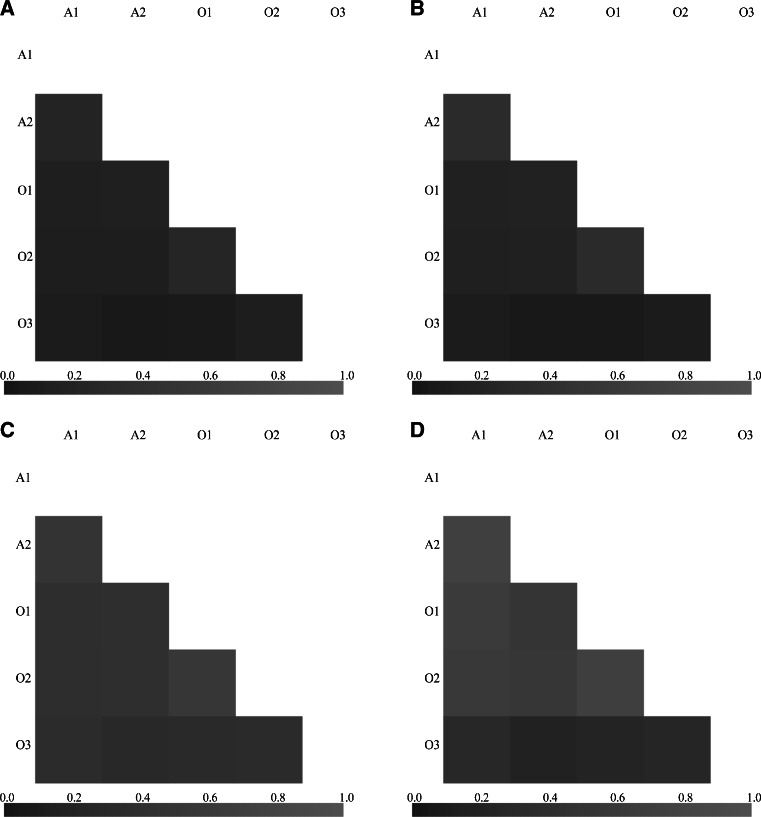
Community distance heatmaps. Darker shade means greater distance (lower similarity). *Light grey* small distances (greater similarity), *dark grey*—greater distances (lower similarity). Heatmaps based on Bray-Curtis dissimilarity **a** at 0.03 dissimilarity level and **b** at 0.10 dissimilarity level; Heatmaps based on Morisita-Horn dissimilarity **c** at 0.03 dissimilarity level and **d** at 0.10 dissimilarity level

To measure the “overlap” between the communities in the samples, an abundance-based index (Morisita-Horn—M-H), as well as an incidence-based one (Bray-Curtis—B-C) were used to calculate distances and community similarity heatmaps were generated (Fig. 4). At both clustering levels, the B-C similarities were smaller than the M-H ones, indicating that the structures of the abundant OTUs subcommunities were more similar than the rare OTUs communities structures. In other words, the rare OTUs were unique for each sample, while the abundant ones were more likely shared. Regardless of the clustering level and the similarity measure, O1 and O2 appeared to be the most similar samples, A1 and A2 formed another pair, while O3 showed little similarity to other ones.

**Fig. 5:**
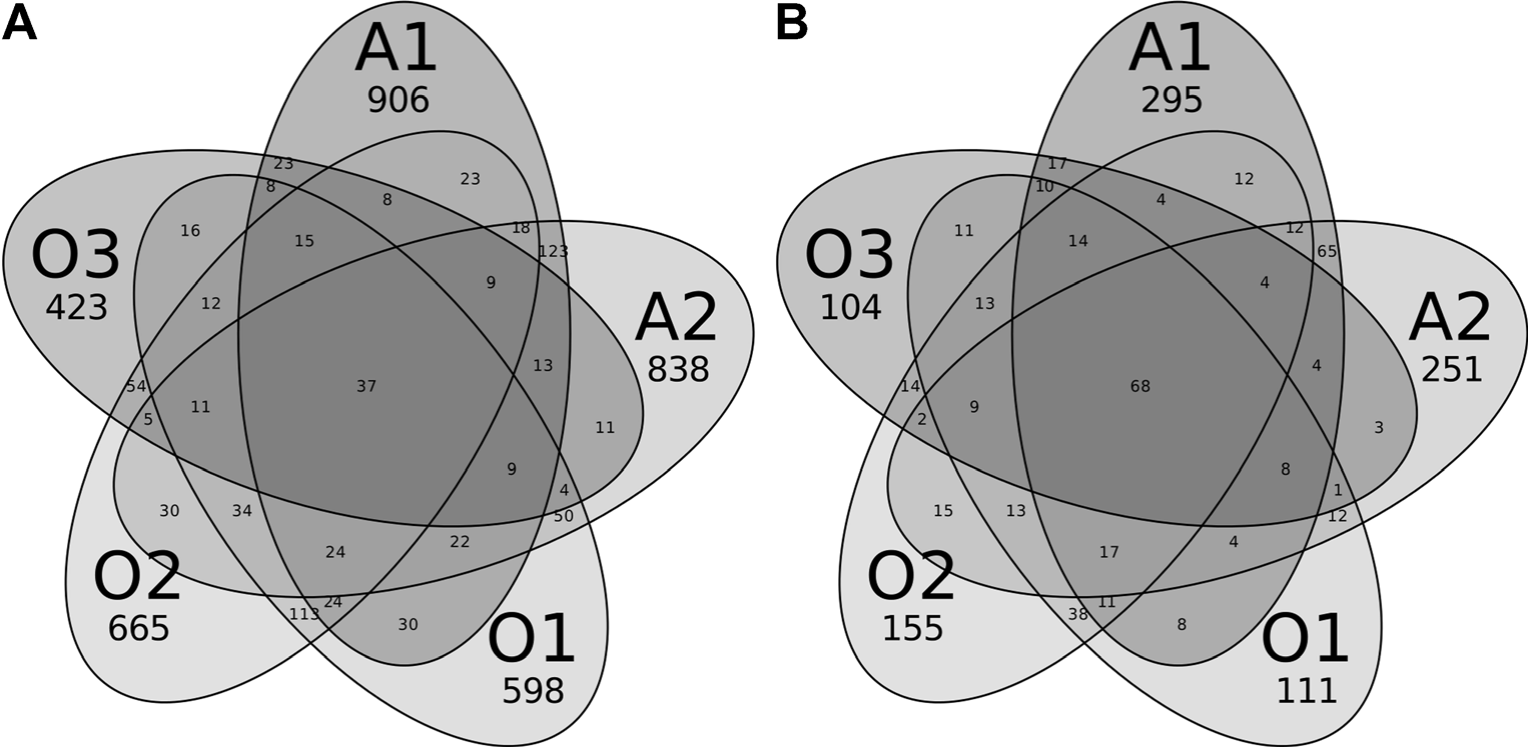
Venn diagrams of shared OTUs. **a** 0.03 dissimilarity level, **b** 0.10 dissimilarity level

The community overlaps were depicted as Venn diagrams (Fig. 5). The results corroborated analyzes of the M-H and B-C similarities presented above: O1 and O2 shared almost identical number of OTUs as A1 and A2. Surprisingly, in spite of differences in the physicochemical features and the plant vegetation between sampling sites, many of the OTUs were shared by all five samples, particularly at the 0.10 clustering level (37 and 68 at 0.03 and 0.10 level, respectively). The percentage of sequences belonging to these OTUs reached 12 % at the 0.03 level and almost 60 % at the 0.10 level.

The statistical significance of differences in community structure were assessed via the parsimony and weighted UniFrac tests. Both methods showed that all the pairs of samples differed significantly (*p* < 0.001).

### Identification of OTUs Shared by all Samples—“Core Microbiome”

To learn which taxons were shared, consensus taxonomy was constructed for each OTU and the ones present in all the samples were identified with an in-house developed Perl script.

At the 0.03 level, the shared OTUs belonged mainly to Alphaproteobacteria (Table S3) and Acidobacteria, with KD4-96 (Chloroflexi), Actinobacteria, and Betaproteobacteria being less frequent. The largest shared OTUs were Otu5473 (an uncultured member of Xanthobacteraceae), Otu5214 (*Sphingomonas*), and Otu4909 (*Candidatus Solibacter*).

At the 0.10 level, the picture was similar (Table S4); however, members of the Bacteroidetes phylum (order Sphingomonadales) were more numerous than Chloroflexi. When a definition of core microbiome given in “Methods” was used, at the 0.03 level, there were four “core” OTUs (Table 7), two belonging to Alphaproteobacteria (genera *Sphingomonas* and *Holospora*), one to Acidobacteria (unclassified Acidobacteriaceae) and one to Chloroflexi (members of KD4-96). At the higher level, there were 21 core OTUs (Table 8), coming mostly from Sphingobacteria (mainly *Flexibacter* and members of Chitinophagaceae), Alphaproteobacteria (members of Sphingomonadaceae and Rhodospirillales), KD4-96 (phylum Chloroflexi), and Betaproteobacteria (members of Nitrosomonadaceae, Comamonadaceae, TRA3-20 and SC-I-84).

**Table 7:** Consensus taxonomies for 0.03 level core microbiome OTUs

	A1	A2	O1	O2	O3	E^a^	Total	Taxonomy
Otu4707	4	3	4	6	6	0,979	23	Proteobacteria; Alphaproteobacteria; Rickettsiales; Holosporaceae; Holospora;
Otu5214	13	26	24	36	41	0,960	140	Proteobacteria; Alphaproteobacteria; Sphingomonadales; Sphingomonadaceae; Sphingomonas;
Otu3722	11	3	12	4	9	0,925	39	Chloroflexi;KD4-96;
Otu4868	6	17	18	15	3	0,903	59	Acidobacteria; Acidobacteria; Acidobacteriales; Acidobacteriaceae;

The numbers of reads in each sample and the total number are reported

^a^Shannon evenness showing how evenly the reads were distributed among samples

**Table 8:** Consensus taxonomies for 0.10 level core microbiome OTUs

	A1	A2	O1	O2	O3	E^a^	Total	Taxonomy
Otu1237	4	8	9	9	8	0.979	38	WCHB1-60;
Otu1558	15	6	8	7	6	0.956	42	Proteobacteria; Gammaproteobacteria; Legionellales; Coxiellaceae; Aquicella;
Otu1464	9	21	3	16	12	0.912	61	Proteobacteria; Deltaproteobacteria; Myxococcales; Nannocystineae; Haliangiaceae; Haliangium;
Otu1410	11	10	9	7	4	0.969	41	Proteobacteria; Deltaproteobacteria;GR-WP33-30;uncultured;
Otu1537	21	3	13	20	14	0.921	71	Proteobacteria; Betaproteobacteria; TRA3-20;
Otu1534	11	22	7	10	15	0.953	65	Proteobacteria; Betaproteobacteria;SC-I-84;
Otu1572	41	21	23	25	53	0.957	163	Proteobacteria; Betaproteobacteria; Nitrosomonadales; Nitrosomonadaceae;uncultured;
Otu1489	11	19	18	4	6	0.909	58	Proteobacteria; Betaproteobacteria; Burkholderiales; Comamonadaceae;
Otu1428	37	46	36	74	48	0.977	241	Proteobacteria; Alphaproteobacteria; Sphingomonadales; Sphingomonadaceae;
Otu1248	10	5	3	5	13	0.919	36	Proteobacteria; Alphaproteobacteria; Rickettsiales; Holosporaceae; Holospora;
Otu1434	35	38	45	36	8	0.938	162	Proteobacteria; Alphaproteobacteria; Rhodospirillales;
Otu1574	7	18	13	26	13	0.948	77	Proteobacteria; Alphaproteobacteria; Rhodospirillales;
Otu1524	12	23	19	19	3	0.916	76	Proteobacteria; Alphaproteobacteria; Rhizobiales; Methylocystaceae;uncultured;
Otu1557	10	13	18	17	3	0.926	61	Proteobacteria; Alphaproteobacteria; Caulobacterales; Caulobacteraceae;
Otu1531	7	4	4	4	3	0.973	22	Chloroflexi;TK10;
Otu1522	28	19	11	14	6	0.928	78	Chloroflexi;S085;
Otu1594	101	56	45	59	55	0.973	316	Chloroflexi;KD4-96;
Otu1504	20	20	10	20	25	0.977	95	Candidate_division_TM7;
Otu1589	74	11	96	61	58	0.915	300	Bacteroidetes; Sphingobacteria; Sphingobacteriales; Cytophagaceae; Flexibacter;
Otu1573	23	79	79	140	40	0.902	361	Bacteroidetes; Sphingobacteria; Sphingobacteriales; Chitinophagaceae;
Otu1470	42	109	90	72	54	0.966	367	Acidobacteria; Acidobacteria; Acidobacteriales; Acidobacteriaceae; Candidatus_Solibacter;

The numbers of reads in each sample and total number are reported

^a^Shannon’s evenness showing how evenly the reads were distributed among samples

## Discussion

In spite of the high level of soils contamination with Pb and Zn, the species richness, diversity, and evenness of bacterial communities under study were similar or slightly lower than those found in studies of unpolluted soils (e.g., [16, 17]). Hur and coworkers [18] found much lower numbers of OTUs in Cd, Pb, and Zn polluted soils (less than 300 observed OTUs at the 0.03 clustering level); however, the soils they analyzed were much more contaminated with Cd (>300 mg kg^−1^) and less with Zn. In spite of the small number of samples, we found a significant negative correlation between bioavailable Zn concentration and all measures of species richness, Shannon’s diversity as well as evenness, which was also similar to Hur’s results [18]. Interestingly, Berg and colleagues [48] found that a long term Cu exposure changes the bacterial community structure, but not its diversity and species richness, demonstrating that various metals influence the communities differently. Similar observations were made by Chodak et al. [20].

Other metals did not correlate with the diversity variables, which might have been caused by the low concentration (cadmium) or the relatively low toxicity (chromium and lead). We did not measure Cr(VI) level, which is the most toxic species of this metal, but the lack of correlation with diversity and richness might indicate that the concentration of chromates was low in our samples. Sheik and coworkers [20] found that Cr(VI) significantly impacted both the diversity and the community structure, lowering the former as well as the species richness.

Interestingly, the plant species richness did not seem to correlate with the bacterial diversity and richness. However, it might be hypothesized that specific plant species influenced these parameters. Which species did that and the kind of the influence remains to be elucidated.

The species richness and the diversity was higher in the Alwernia soils than in the Olkusz ones. The reason for this might be the soil texture, as there are evidences that higher clay content promotes bacterial diversity [49, 50], or OM and Cr, which seem to be positively correlated in our samples.

The samples were taken in a way to avoid sampling rhizosphere communities (depth 5–30 cm, roots removed with soil sticking to them); however, in the case of the Olkusz ones, it was more difficult (sandy soils, rhizosphere not sticking to roots), which could have caused certain amount of the rhizospheric soil to contaminate the samples. Certain reports [51] showed that the rhizosphere harbored less species than the bulk soil. It is also possible that plants that were common at the Olkusz sites (e.g., *Pinus sylvestris*) but absent from the Alwernia ones favored specific communities with lower diversity. There are papers showing that plant species together with the soil type determine the shape of the microbial community in the rhizosphere [52, 53].

The bacterial communities at the level of phyla were similar in all our samples, in spite of the distance separating the sampling sites and the differences in other parameters such as the plant cover and the contamination. They were generally similar to those found in other soils analyzed by the pyrosequencing-based methods, even though different primer systems were used. Proteobacteria were found to be the dominant phylum both in grassland (e.g., [16–18]) and forest soils [16], just as in the Olkusz and Alwernia samples. Other abundant phyla were Actinobacteria and Acidobacteria followed by Bacteroidetes and Firmicutes. The three first phyla were also present in high numbers in our samples; however, the last one was relatively rare. Thus, the level of phylum does not seem to be adequate for assessing differences in soil bacterial communities. The same, to a lesser extent, applies also to the class level. Lower levels should therefore be used if the effect of heavy metals is to be demonstrated. In our case, both the levels chosen (0.03—roughly species, 0.10—family) allowed the demonstration of Zn influence on bacterial communities. It seems that the lower the level, the more specific effects can be seen, but on the other hand going below the 0.03 dissimilarity is difficult due to the sequencing noise, therefore constructing the OTUs at the 0.03 level is now the best choice. For large studies, it might be that higher levels would be more appropriate, as the variability at the strain or species level may be too high.

As we were interested in defining the core microbiome of our samples, we decided to use the level of species and families (0.03 and 0.10 OTUs, respectively). We chose to assign evenly distributed OTUs with over two reads in each sample to the core microbiome to lower the probability that rare sequences that were present in one or more samples purely by chance (e.g., due to the erroneous reading of a MID sequence) would be taken as shared. Core microbiomes were to date determined for various parts of human body in the Human Microbiome Project (e.g., [46]) and outside of HMP [54]; however, to our knowledge, this is the first attempt to define the core microbiome of soil samples. Our samples’ core microbiome appeared to be fairly large and even at the 0.03 level, a single OTU (Otu5214, *Sphingomonas*) constituted over 0.5 % of total reads number in each sample. At the level of 0.10 dissimilarity, there were six such OTUs, some of them present at the level over 1.71 %. In the case of human body core microbiomes, they were impossible to define at low phylogenetic levels and no abundant OTUs (over 0.5 % of reads from each sample) were universally shared [54]. As our samples were diverse in terms of physicochemical properties except for the Zn contamination, it seems that the core OTUs represent bacteria that are adapted to heavy metals, albeit various ones (Zn and Pb in Olkusz samples, while Zn and Cr in Alwernia). It would be interesting to see if more geographically distant samples also share so many phylotypes at low phylogenetic level and if the core genes set could be defined. The latter question could be addressed by deep sequencing of metagenomic DNA, assembly, and analysis of genes encoded on the assembled contigs.

In conclusion, our work showed that OTUs constructed at the 0.03 and 0.10 levels allow assessing the influence of heavy metals on both bacterial diversity and community structure. Zn turned out to be the factor that had the greatest impact on these parameters, other were less important; in particular, no correlation between plant diversity and bacterial diversity was observed. We defined our samples core microbiome comprising *Holospora* and *Sphingomonas* (Alphaproteobacteria) as well as KD4-96 (Chloroflexi) and an unclassified member of Acidobacteriaceae at the species (0.03) level and Cytophagaceae, Chitinophagaceae, Acidobacteriaceae, KD4-96, Sphingomonadaceae, and Nitrosomonadaceae at the family level (0.10).

## Electronic supplementary material

Below is the link to the electronic supplementary material.

ESM 1 (DOC 1249 kb)
