# Supplementary material for: 16S rDNA Pyrosequencing Analysis of Bacterial Community in Heavy Metals Polluted Soils
Source: Microb Ecol. 2014 Jan 9;67(3):635–47. doi: 10.1007/s00248-013-0344-7 (PMC3962847; doi:10.1007/s00248-013-0344-7)
Supplement: Supplementary file 1 — (DOC 1249 kb) [file 248_2013_344_MOESM1_ESM.doc]

Supplementary Materials

16S rDNA pyrosequencing analysis of bacterial community in heavy metals polluted soils

1. **Parameters used in pyrosequencing reads analysis**

Flowgrams extraction: sffinfo at default settings.

Flowgrams trimming: trim.flows pdiffs=2, bdiffs=1.

Denoising flowgrams: shhh.flows at default settings, lookup=LookUp_Titanium.pat.

Removing primer and MID sequence: trim.seqs pdiffs=2, bdiffs=1.

Dereplication: unique.seqs at default settings.

Alignment: align.seqs reference=silva.bacteria.fasta.

Screening reads: screen.seqs start=6428, end=16000.

Filtering: filter.seqs vertical=T, trump=.

Chimera identification: chimera.uchime template=self.

Pre-clustering: pre.cluster diffs=2.

Distance matrix calculation: dist.seqs cutoff=1.

OTUs construction: cluster method=average

Shared OTUs file construction: make.shared label=0.03-0.10

Reads classification: classify.seqs reference=nogap.bacteria.fasta, taxonomy=silva.bacteria.silva.tax, cutoff=80.

**2. Preparation of averaged shared OTU table**

The sub.sample command of MOTHUR was used to draw five random subsamples from the final set of reads (high quality, non-chimeric):

sub.sample(fasta=ao.final.fasta, name=ao.final.names, group=ao.final.groups, size=1500, persample=T)

Read names in the resulting files were modified using search and replace function of the vi editor:

vi ao.final.subsampleX.fasta # X is the number of subsample

:%s/>/>X_/

:qw

vi ao.final.subsampleX.groups

:%s/^/^X_/

:%s/$/_X$/

:qw

vi ao.final.subsampleX.names

:%s/^/^X_/

:%s/\t/\tX_/

:%s/\,/\,X_/g

:qw

The reads from 5 subsamples were then combined:

cat ao.subsample*.fasta > ao.all.fasta

cat ao.subsample*.names > ao.all.names

cat ao.subsample*.groups > ao.all.groups

The reads were dereplicated (unique.seqs), distance matrix was calculated (dist.seqs), OTUs constructed (cluster) and shared OTUs table made with make.shared.

The average shared OTUs tables were constructed in R. They comprised OTUs represented by more than 50 sequences in the entire set (i.e. on average 2 per bootstrapped subsample).

First, the OTUs with more than 50 reads were selected:

shared003over50 ← shared003[,colSums(shared003)>50]

Then column means rounded to the nearest integer were calculated for submatrices comprising five subsamples of the same sample:

X ← round(colMeans(shared003over50[grep("X", row.names(shared003over50)),])) # X is the name of sample (Alwernia1, Alwernia2, Olkusz1, Olkusz2, Olkusz3)

Then the obtained rows were combined into the final matrix:

shared003over50averaged ← rbind(Alwernia1, Alwernia2, Olkusz1, Olkusz2, Olkusz3)

**Table S1. Results of reads classification at phylum, class and family level.**

| **Phyla** | | **Total** | **A1** | **A2** | **O1** | **O2** | **O3** |
| --- | --- | --- | --- | --- | --- | --- | --- |
|  | Proteobacteria | 38.0 | 44.5 | 38.9 | 36.7 | 27.5 | 49.5 |
|  | Actinobacteria | 14.6 | 4.9 | 7.0 | 23.5 | 19.6 | 5.6 |
|  | Acidobacteria | 13.9 | 14.6 | 24.8 | 11.9 | 9.5 | 13.8 |
|  | Bacteroidetes | 9.6 | 7.5 | 6.3 | 10.4 | 14.7 | 5.3 |
|  | Chloroflexi | 6.7 | 11.7 | 6.4 | 4.2 | 8.5 | 5.6 |
|  | Gemmatimonadetes | 5.8 | 4.6 | 5.3 | 5.3 | 4.2 | 9.8 |
|  | Verrucomicrobia | 2.8 | 2.8 | 1.7 | 1.9 | 6.2 | 0.7 |
|  | Firmicutes | 1.7 | 1.5 | 2.0 | 1.3 | 2.7 | 0.7 |
|  | Candidate_division_TM7 | 1.1 | 1.0 | 0.8 | 0.9 | 1.2 | 1.7 |
|  | Nitrospirae | 0.7 | 1.5 | 1.9 | 0.3 | 0.6 | 0.3 |
|  | Candidate_division_TM6 | 0.7 | 0.1 | 0.7 | 0.6 | 0.3 | 1.4 |
|  | Planctomycetes | 0.5 | 1.1 | 0.2 | 0.3 | 0.7 | 0.5 |
|  | Candidate_division_WS3 | 0.5 | 0.6 | 1.4 | 0.2 | 0.0 | 0.7 |
|  | Chlorobi | 0.5 | 0.5 | 0.1 | 0.2 | 0.8 | 0.6 |
|  | Lentisphaerae | 0.4 | 0.2 | 0.2 | 0.2 | 0.1 | 1.7 |
|  | WCHB1-60 | 0.4 | 0.3 | 0.6 | 0.3 | 0.4 | 0.4 |
|  | Candidate_division_TG-1 | 0.4 | 0.2 | 0.3 | 0.6 | 0.4 | 0.2 |
|  | Candidate_division_OP10 | 0.3 | 0.4 | 0.1 | 0.0 | 0.6 | 0.5 |
|  | Candidate_division_OD1 | 0.3 | 0.3 | 0.0 | 0.1 | 0.4 | 0.6 |
|  | Fibrobacteres | 0.2 | 0.4 | 0.0 | 0.1 | 0.6 | 0.0 |
|  | Cyanobacteria | 0.2 | 0.5 | 0.2 | 0.2 | 0.2 | 0.1 |
|  | TA06 | 0.2 | 0.0 | 0.1 | 0.5 | 0.0 | 0.0 |
|  | Chlamydiae | 0.1 | 0.3 | 0.2 | 0.2 | 0.0 | 0.1 |
|  | Others (<20) | 0.5 | 0.8 | 0.7 | 0.3 | 0.7 | 0.2 |
| **Classes** | | | | | | | |
|  | Alphaproteobacteria | 14.9 | 16.9 | 13.4 | 22.1 | 13.0 | 6.0 |
|  | Actinobacteria | 14.6 | 4.9 | 7.0 | 23.5 | 19.6 | 5.6 |
|  | Acidobacteria | 11.9 | 11.2 | 20.6 | 10.2 | 8.0 | 13.4 |
|  | Gammaproteobacteria | 8.7 | 8.5 | 4.4 | 3.8 | 4.2 | 25.9 |
|  | Sphingobacteria | 8.5 | 5.5 | 4.9 | 9.7 | 12.9 | 5.0 |
|  | Betaproteobacteria | 8.3 | 10.4 | 6.3 | 7.1 | 5.0 | 15.1 |
|  | Deltaproteobacteria | 5.9 | 7.6 | 14.8 | 3.7 | 5.1 | 2.5 |
|  | Gemmatimonadetes | 5.8 | 4.6 | 5.3 | 5.3 | 4.2 | 9.8 |
|  | KD4-96 | 2.9 | 4.2 | 2.2 | 1.8 | 4.2 | 2.5 |
|  | OPB35 | 1.9 | 2.1 | 1.0 | 0.9 | 4.5 | 0.4 |
|  | Holophagae | 1.6 | 3.1 | 3.1 | 1.6 | 1.2 | 0.3 |
|  | S085 | 1.3 | 2.0 | 1.5 | 1.0 | 1.8 | 0.5 |
|  | unclassified Candidate division TM7 | 1.1 | 1.0 | 0.8 | 0.9 | 1.2 | 1.7 |
|  | Bacilli | 1.1 | 0.2 | 0.4 | 1.2 | 2.3 | 0.4 |
|  | Flavobacteria | 1.1 | 1.8 | 1.3 | 0.6 | 1.7 | 0.3 |
|  | Spartobacteria | 0.9 | 0.3 | 0.6 | 0.9 | 1.6 | 0.3 |
|  | Nitrospira | 0.7 | 1.5 | 1.9 | 0.3 | 0.6 | 0.3 |
|  | TK10 (Chloroflexi) | 0.7 | 0.9 | 0.2 | 0.3 | 0.4 | 2.1 |
|  | unclassified Candidate division TM6 | 0.7 | 0.1 | 0.7 | 0.6 | 0.3 | 1.4 |
|  | Clostridia | 0.5 | 1.3 | 1.5 | 0.1 | 0.3 | 0.3 |
|  | unclassified Candidate division WS3 | 0.5 | 0.6 | 1.4 | 0.2 | 0.0 | 0.7 |
|  | Chlorobia | 0.5 | 0.5 | 0.1 | 0.2 | 0.8 | 0.6 |
|  | Lentisphaeria | 0.4 | 0.2 | 0.2 | 0.2 | 0.1 | 1.7 |
|  | Chloroflexi | 0.4 | 1.3 | 0.3 | 0.2 | 0.4 | 0.1 |
|  | unclassified WCHB1-60 | 0.4 | 0.3 | 0.6 | 0.3 | 0.4 | 0.4 |
|  | Anaerolineae | 0.4 | 1.5 | 0.8 | 0.1 | 0.2 | 0.1 |
|  | Lineage_IV (Candidate division TG1) | 0.4 | 0.2 | 0.3 | 0.6 | 0.4 | 0.2 |
|  | RB25 (Acidobacteria) | 0.3 | 0.3 | 1.1 | 0.1 | 0.4 | 0.1 |
|  | unclassified Candidate division OP10 | 0.3 | 0.4 | 0.1 | 0.0 | 0.6 | 0.5 |
|  | GIF3 (Chloroflexi) | 0.3 | 0.4 | 0.7 | 0.1 | 0.4 | 0.0 |
|  | Planctomycetacia | 0.3 | 0.6 | 0.1 | 0.3 | 0.3 | 0.2 |
|  | unclassified Candidate division OD1 | 0.3 | 0.3 | 0.0 | 0.1 | 0.4 | 0.6 |
|  | Thermomicrobia | 0.3 | 0.1 | 0.2 | 0.2 | 0.5 | 0.1 |
|  | Fibrobacteria | 0.2 | 0.4 | 0.0 | 0.1 | 0.6 | 0.0 |
|  | Caldilineae | 0.2 | 0.6 | 0.4 | 0.1 | 0.2 | 0.1 |
|  | unclassified TA06 | 0.2 | 0.0 | 0.1 | 0.5 | 0.0 | 0.0 |
|  | Chlamydiae | 0.1 | 0.3 | 0.2 | 0.2 | 0.0 | 0.1 |
|  | SHA-26 (Chloroflexi) | 0.1 | 0.0 | 0.0 | 0.1 | 0.3 | 0.0 |
|  | SAR202 (Chloroflexi) | 0.1 | 0.2 | 0.0 | 0.2 | 0.0 | 0.1 |
|  | Phycisphaerae | 0.1 | 0.1 | 0.1 | 0.0 | 0.1 | 0.2 |
|  | JTB23 (Proteobacteria) | 0.1 | 0.9 | 0.0 | 0.0 | 0.0 | 0.0 |
|  | Others (<20) | 1.1 | 2.5 | 1.3 | 0.5 | 1.6 | 0.0 |
| **Orders** | | | | | | | |
|  | Acidobacteriales | 11.9 | 11.2 | 20.6 | 10.2 | 8.0 | 13.4 |
|  | Rhizobiales | 9.4 | 10.5 | 7.6 | 16.7 | 6.9 | 2.0 |
|  | Sphingobacteriales | 8.5 | 5.5 | 4.9 | 9.7 | 12.9 | 5.0 |
|  | Gemmatimonadales | 5.7 | 4.6 | 5.3 | 5.2 | 4.2 | 9.6 |
|  | Actinomycetales | 4.1 | 1.7 | 3.5 | 6.3 | 5.4 | 0.9 |
|  | AKIW543 (Actinobacteria) | 3.7 | 0.8 | 0.6 | 7.1 | 4.1 | 2.1 |
|  | Acidimicrobiales | 3.7 | 2.0 | 2.0 | 5.6 | 4.6 | 1.9 |
|  | Nitrosomonadales | 3.4 | 3.9 | 3.0 | 2.9 | 1.8 | 6.4 |
|  | Xanthomonadales | 3.0 | 3.8 | 2.5 | 2.4 | 2.1 | 5.1 |
|  | unclassified KD4-96 (Chloroflexi) | 2.9 | 4.2 | 2.2 | 1.8 | 4.2 | 2.5 |
|  | Thiotrichales | 2.8 | 0.4 | 0.3 | 0.1 | 0.4 | 13.9 |
|  | Myxococcales | 2.6 | 2.4 | 3.7 | 1.8 | 3.8 | 1.4 |
|  | Rhodospirillales | 2.4 | 2.8 | 2.6 | 3.1 | 2.5 | 1.0 |
|  | Solirubrobacterales | 2.1 | 0.2 | 0.5 | 2.9 | 4.2 | 0.5 |
|  | Burkholderiales | 1.9 | 2.6 | 1.7 | 3.1 | 1.3 | 0.7 |
|  | unclassified OPB35 (Verrucomicrobia) | 1.9 | 2.1 | 1.0 | 0.9 | 4.5 | 0.4 |
|  | Desulfuromonadales | 1.8 | 2.7 | 8.8 | 0.2 | 0.3 | 0.2 |
|  | Sphingomonadales | 1.8 | 1.5 | 1.8 | 1.5 | 2.1 | 1.9 |
|  | unclassified S085 (Chloroflexi) | 1.3 | 2.0 | 1.5 | 1.0 | 1.8 | 0.5 |
|  | unclassified Candidate division TM7 | 1.1 | 1.0 | 0.8 | 0.9 | 1.2 | 1.7 |
|  | Flavobacteriales | 1.1 | 1.8 | 1.3 | 0.6 | 1.7 | 0.3 |
|  | Bacillales | 1.0 | 0.2 | 0.4 | 1.1 | 2.0 | 0.4 |
|  | Hydrogenophilales | 1.0 | 0.1 | 0.1 | 0.0 | 0.3 | 4.7 |
|  | Legionellales | 0.9 | 1.6 | 0.6 | 0.5 | 0.9 | 1.4 |
|  | GR-WP33-30 (Deltaproteobacteria) | 0.9 | 1.3 | 1.5 | 1.0 | 0.6 | 0.2 |
|  | 32-20 (Holophagae) | 0.8 | 1.2 | 2.0 | 0.9 | 0.4 | 0.0 |
|  | Rhodocyclales | 0.8 | 2.2 | 0.3 | 0.3 | 0.1 | 2.1 |
|  | MB-A2-108 (Actinobacteria) | 0.8 | 0.2 | 0.4 | 1.5 | 0.8 | 0.1 |
|  | Nitrospirales | 0.7 | 1.5 | 1.9 | 0.3 | 0.6 | 0.3 |
|  | unclassified TK10 (Chloroflexi) | 0.7 | 0.9 | 0.2 | 0.3 | 0.4 | 2.1 |
|  | 196up (Gammaproteobacteria) | 0.7 | 0.1 | 0.1 | 0.1 | 0.0 | 3.2 |
|  | unclassified Candidate division TM7 | 0.7 | 0.1 | 0.7 | 0.6 | 0.3 | 1.4 |
|  | DA101 (Spartobacteria) | 0.6 | 0.0 | 0.4 | 0.8 | 1.4 | 0.0 |
|  | Caulobacterales | 0.6 | 0.8 | 0.7 | 0.6 | 1.0 | 0.2 |
|  | SC-I-84 (Betaproteobacteria) | 0.5 | 0.6 | 1.1 | 0.4 | 0.4 | 0.5 |
|  | iii1-8 (Holophagae) | 0.5 | 1.7 | 0.4 | 0.2 | 0.6 | 0.3 |
|  | TRA3-20 (Betaproteobacteria) | 0.5 | 0.6 | 0.1 | 0.5 | 0.7 | 0.5 |
|  | Rickettsiales | 0.5 | 0.7 | 0.5 | 0.2 | 0.5 | 0.8 |
|  | Clostridiales | 0.5 | 1.0 | 1.5 | 0.1 | 0.2 | 0.3 |
|  | unclassified candidate division WS3 | 0.5 | 0.6 | 1.4 | 0.2 | 0.0 | 0.7 |
|  | Chlorobiales | 0.5 | 0.5 | 0.1 | 0.2 | 0.8 | 0.6 |
|  | WCHB1-25 (Lentisphaeria) | 0.4 | 0.1 | 0.2 | 0.2 | 0.0 | 1.6 |
|  | Pseudomonadales | 0.4 | 1.5 | 0.3 | 0.3 | 0.3 | 0.2 |
|  | Chloroflexales | 0.4 | 1.3 | 0.3 | 0.2 | 0.4 | 0.1 |
|  | unclassified WCHB1-60 | 0.4 | 0.3 | 0.6 | 0.3 | 0.4 | 0.4 |
|  | Anaerolineales | 0.4 | 1.5 | 0.8 | 0.1 | 0.2 | 0.1 |
|  | unclassified Lineage IV (Candidate division TG1) | 0.4 | 0.2 | 0.3 | 0.6 | 0.4 | 0.2 |
|  | unclassified RB25 (Acidobacteria) | 0.3 | 0.3 | 1.1 | 0.1 | 0.4 | 0.1 |
|  | unclassified Candidate division OP10 | 0.3 | 0.4 | 0.1 | 0.0 | 0.6 | 0.5 |
|  | unclassified GIF3 (Chloroflexi) | 0.3 | 0.4 | 0.7 | 0.1 | 0.4 | 0.0 |
|  | Planctomycetales | 0.3 | 0.6 | 0.1 | 0.3 | 0.3 | 0.2 |
|  | SJA-36 (Holophagae) | 0.3 | 0.1 | 0.6 | 0.5 | 0.1 | 0.0 |
|  | Chromatiales | 0.3 | 0.5 | 0.2 | 0.1 | 0.2 | 0.5 |
|  | unclassified candidate division OD1 | 0.3 | 0.3 | 0.0 | 0.1 | 0.4 | 0.6 |
|  | Fibrobacterales | 0.2 | 0.4 | 0.0 | 0.1 | 0.6 | 0.0 |
|  | Caldilineales | 0.2 | 0.6 | 0.4 | 0.1 | 0.2 | 0.1 |
|  | Bdellovibrionales | 0.2 | 0.0 | 0.1 | 0.2 | 0.2 | 0.3 |
|  | Thiohalophilus | 0.2 | 0.0 | 0.0 | 0.0 | 0.0 | 0.9 |
|  | Desulfurellales | 0.2 | 0.2 | 0.1 | 0.2 | 0.2 | 0.3 |
|  | AKYG1722 (Thermomicrobia) | 0.2 | 0.1 | 0.1 | 0.2 | 0.3 | 0.0 |
|  | NKB5 (Gammaproteobacteria) | 0.2 | 0.3 | 0.1 | 0.1 | 0.2 | 0.2 |
|  | unclassified TA06 | 0.2 | 0.0 | 0.1 | 0.5 | 0.0 | 0.0 |
|  | Desulfovibrionales | 0.2 | 0.4 | 0.1 | 0.3 | 0.0 | 0.0 |
|  | Chlamydiales | 0.1 | 0.3 | 0.2 | 0.2 | 0.0 | 0.1 |
|  | Neisseriales | 0.1 | 0.2 | 0.0 | 0.0 | 0.3 | 0.2 |
|  | unclassified SHA26 (Chloroflexi) | 0.1 | 0.0 | 0.0 | 0.1 | 0.3 | 0.0 |
|  | Rhodobacterales | 0.1 | 0.3 | 0.1 | 0.1 | 0.1 | 0.1 |
|  | unclassified SAR202 (Chloroflexi) | 0.1 | 0.2 | 0.0 | 0.2 | 0.0 | 0.1 |
|  | Desulfobacterales | 0.1 | 0.3 | 0.3 | 0.0 | 0.0 | 0.0 |
|  | unclassified JTB23 (Proteobacteria) | 0.1 | 0.9 | 0.0 | 0.0 | 0.0 | 0.0 |
|  | Lactobacillales | 0.1 | 0.0 | 0.0 | 0.1 | 0.3 | 0.0 |
|  | Coriobacteriales | 0.1 | 0.0 | 0.1 | 0.1 | 0.2 | 0.1 |
|  | Others (<20) | 1.9 | 4.0 | 2.2 | 0.8 | 2.4 | 1.5 |
| **Families** | | | | | | | |
|  | Acidobacteriaceae | 11.9 | 11.2 | 20.6 | 10.2 | 8.0 | 13.4 |
|  | Gemmatimonadaceae | 5.7 | 4.6 | 5.3 | 5.2 | 4.2 | 9.6 |
|  | Chitinophagaceae | 4.0 | 1.2 | 3.5 | 3.8 | 7.5 | 2.0 |
|  | Acidimicrobiales | 3.7 | 2.0 | 2.0 | 5.6 | 4.6 | 1.9 |
|  | Nitrosomonadaceae | 3.3 | 3.8 | 2.7 | 2.9 | 1.7 | 6.4 |
|  | Xanthobacteraceae | 3.0 | 2.3 | 2.6 | 5.6 | 2.6 | 0.3 |
|  | unclassified KD4-96 (Chloroflexi) | 2.9 | 4.2 | 2.2 | 1.8 | 4.2 | 2.5 |
|  | Cytophagaceae | 2.8 | 3.1 | 0.4 | 4.1 | 2.9 | 2.5 |
|  | Thiotrichaceae | 2.2 | 0.3 | 0.0 | 0.0 | 0.0 | 11.6 |
|  | Sinobacteraceae | 1.9 | 2.7 | 1.2 | 1.9 | 1.3 | 3.0 |
|  | unclassified OPB35 (Verrucomicrobia) | 1.9 | 2.1 | 1.0 | 0.9 | 4.5 | 0.4 |
|  | Solirubrobacteriaceae | 1.8 | 0.1 | 0.4 | 2.3 | 3.7 | 0.5 |
|  | Bradyrhizobiaceae | 1.7 | 0.2 | 0.2 | 5.0 | 0.4 | 0.0 |
|  | Geobacteraceae | 1.6 | 2.5 | 7.9 | 0.2 | 0.3 | 0.1 |
|  | Sphingomonadaceae | 1.6 | 1.1 | 1.3 | 1.4 | 1.9 | 1.8 |
|  | unclassified S085 (Chloroflexi) | 1.3 | 2.0 | 1.5 | 1.0 | 1.8 | 0.5 |
|  | Methylocystaceae | 1.3 | 2.0 | 1.5 | 1.4 | 1.5 | 0.2 |
|  | unclassified candidate division TM7 | 1.1 | 1.0 | 0.8 | 0.9 | 1.2 | 1.7 |
|  | Nannocystineae | 1.1 | 0.8 | 1.6 | 0.6 | 1.8 | 0.7 |
|  | Xanthomonadaceae | 1.1 | 1.1 | 1.3 | 0.5 | 0.8 | 2.1 |
|  | Comamonadaceae | 1.0 | 2.2 | 1.5 | 1.2 | 0.5 | 0.3 |
|  | Haliangiaceae | 1.0 | 0.7 | 1.4 | 0.6 | 1.7 | 0.6 |
|  | Hydrogenophilaceae | 1.0 | 0.1 | 0.1 | 0.0 | 0.3 | 4.7 |
|  | Hyphomicrobiaceae | 1.0 | 1.0 | 0.8 | 1.5 | 0.8 | 0.5 |
|  | Acidimicrobiaceae | 1.0 | 0.5 | 0.6 | 1.4 | 1.1 | 0.6 |
|  | Sphingomonadaceae | 0.9 | 0.6 | 0.7 | 0.8 | 1.2 | 1.3 |
|  | Flavobacteriaceae | 0.8 | 1.5 | 1.3 | 0.5 | 1.0 | 0.2 |
|  | unclassified 32-20 (Holophagae) | 0.8 | 1.2 | 2.0 | 0.9 | 0.4 | 0.0 |
|  | Rhodocyclaceae | 0.8 | 2.2 | 0.3 | 0.3 | 0.1 | 2.1 |
|  | Nitrospiraceae | 0.7 | 1.5 | 1.9 | 0.3 | 0.6 | 0.3 |
|  | unclassified TK10 (Chloroflexi) | 0.7 | 0.9 | 0.2 | 0.3 | 0.4 | 2.1 |
|  | Coxiellaceae | 0.7 | 1.5 | 0.6 | 0.4 | 0.5 | 1.0 |
|  | unclassified 196up (Gammaproteobacteria) | 0.7 | 0.1 | 0.1 | 0.1 | 0.0 | 3.2 |
|  | unclassified candidate division TM7 | 0.7 | 0.1 | 0.7 | 0.6 | 0.3 | 1.4 |
|  | unclassified DA101 (Spartobacteria) | 0.6 | 0.0 | 0.4 | 0.8 | 1.4 | 0.0 |
|  | wr0007 (Rhodospirillales) | 0.6 | 0.3 | 0.6 | 0.6 | 1.0 | 0.2 |
|  | Mycobacteriaceae | 0.6 | 0.3 | 0.1 | 1.1 | 0.8 | 0.1 |
|  | Cystobacterineae | 0.6 | 0.5 | 1.2 | 0.3 | 0.7 | 0.4 |
|  | Rhodospirillaceae | 0.6 | 0.7 | 0.5 | 0.6 | 0.6 | 0.5 |
|  | unclassified SC-I-84 (Betaproteobacteria) | 0.5 | 0.6 | 1.1 | 0.4 | 0.4 | 0.5 |
|  | unclassified iii1-8 (Holophagae) | 0.5 | 1.7 | 0.4 | 0.2 | 0.6 | 0.3 |
|  | Sphingobacteriaceae | 0.5 | 0.1 | 0.2 | 0.7 | 0.9 | 0.1 |
|  | unclassified TRA3-20 (Betaproteobacteria) | 0.5 | 0.6 | 0.1 | 0.5 | 0.7 | 0.5 |
|  | Caulobacteraceae | 0.5 | 0.4 | 0.6 | 0.5 | 0.8 | 0.1 |
|  | Rhodobiaceae | 0.5 | 1.0 | 0.5 | 0.5 | 0.4 | 0.3 |
|  | unclassified candidate division WS3 | 0.5 | 0.6 | 1.4 | 0.2 | 0.0 | 0.7 |
|  | Nocardioidaceae | 0.4 | 0.2 | 0.2 | 0.7 | 0.7 | 0.0 |
|  | unclassified WCHB1-25 (Lentisphaeria) | 0.4 | 0.1 | 0.2 | 0.2 | 0.0 | 1.6 |
|  | Paenibacillaceae | 0.4 | 0.0 | 0.1 | 0.5 | 1.0 | 0.1 |
|  | Acidothermaceae | 0.4 | 0.0 | 0.0 | 1.1 | 0.2 | 0.1 |
|  | Acetobacteraceae | 0.4 | 0.5 | 0.4 | 0.8 | 0.1 | 0.1 |
|  | Piscirickettsiaceae | 0.4 | 0.0 | 0.0 | 0.0 | 0.1 | 1.9 |
|  | Oxalobacteraceae | 0.4 | 0.2 | 0.0 | 1.0 | 0.2 | 0.1 |
|  | unclassified WCHB1-60 | 0.4 | 0.3 | 0.6 | 0.3 | 0.4 | 0.4 |
|  | Sorangiineae | 0.4 | 0.6 | 0.7 | 0.5 | 0.2 | 0.0 |
|  | Anaerolineaceae | 0.4 | 1.5 | 0.8 | 0.1 | 0.2 | 0.1 |
|  | unclassified Lineage IV (Candidate division TG1) | 0.4 | 0.2 | 0.3 | 0.6 | 0.4 | 0.2 |
|  | Pseudonocardiaceae | 0.4 | 0.2 | 0.9 | 0.2 | 0.5 | 0.1 |
|  | Micromonosporaceae | 0.3 | 0.3 | 0.7 | 0.5 | 0.3 | 0.0 |
|  | Conexibacteraceae | 0.3 | 0.1 | 0.1 | 0.6 | 0.5 | 0.1 |
|  | Cystobacteraceae | 0.3 | 0.3 | 0.9 | 0.2 | 0.5 | 0.0 |
|  | unclassified RB25 (Acidobacteria) | 0.3 | 0.3 | 1.1 | 0.1 | 0.4 | 0.1 |
|  | Iamiaceae | 0.3 | 0.3 | 0.1 | 0.3 | 0.6 | 0.2 |
|  | env.OPS_17 (Spartobacteria) | 0.3 | 0.4 | 0.3 | 0.3 | 0.4 | 0.1 |
|  | unclassified candidate division OP10 | 0.3 | 0.4 | 0.1 | 0.0 | 0.6 | 0.5 |
|  | Phyllobacteriaceae | 0.3 | 0.3 | 0.1 | 0.7 | 0.2 | 0.0 |
|  | Sphingobacteriaceae | 0.3 | 0.0 | 0.1 | 0.6 | 0.4 | 0.1 |
|  | MND8 (Rhodospirillales) | 0.3 | 0.6 | 0.2 | 0.5 | 0.2 | 0.0 |
|  | Burkholderiaceae | 0.3 | 0.0 | 0.0 | 0.7 | 0.4 | 0.1 |
|  | Chloroflexaceae | 0.3 | 1.2 | 0.3 | 0.2 | 0.3 | 0.0 |
|  | Pseudomonadaceae | 0.3 | 0.9 | 0.3 | 0.3 | 0.1 | 0.2 |
|  | JG37-AG-15 (Myxococcales) | 0.3 | 0.5 | 0.1 | 0.1 | 0.6 | 0.3 |
|  | Propionibacteriaceae | 0.3 | 0.1 | 0.3 | 0.5 | 0.3 | 0.1 |
|  | Flammeovirgaceae | 0.3 | 0.2 | 0.2 | 0.3 | 0.5 | 0.3 |
|  | unclassified GIF3 (Chloroflexi) | 0.3 | 0.4 | 0.7 | 0.1 | 0.4 | 0.0 |
|  | Planctomycetaceae | 0.3 | 0.6 | 0.1 | 0.3 | 0.3 | 0.2 |
|  | Beijerinckiaceae | 0.3 | 0.1 | 0.1 | 0.7 | 0.1 | 0.1 |
|  | Micrococcaceae | 0.3 | 0.0 | 0.0 | 0.3 | 0.7 | 0.0 |
|  | unclassified SJA-36 (Holophagae) | 0.3 | 0.1 | 0.6 | 0.5 | 0.1 | 0.0 |
|  | unclassified candidate division OD1 | 0.3 | 0.3 | 0.0 | 0.1 | 0.4 | 0.6 |
|  | SJA-28 (Chlorobiales) | 0.2 | 0.3 | 0.1 | 0.1 | 0.6 | 0.0 |
|  | Fibrobacteraceae | 0.2 | 0.4 | 0.0 | 0.1 | 0.6 | 0.0 |
|  | Clostridiaceae | 0.2 | 0.6 | 1.0 | 0.0 | 0.1 | 0.1 |
|  | Frankiaceae | 0.2 | 0.1 | 0.1 | 0.3 | 0.5 | 0.0 |
|  | Caldilineaceae | 0.2 | 0.6 | 0.4 | 0.1 | 0.2 | 0.1 |
|  | Legionellaceae | 0.2 | 0.1 | 0.0 | 0.1 | 0.4 | 0.4 |
|  | MNH4 (Rhodospirillales) | 0.2 | 0.3 | 0.4 | 0.2 | 0.1 | 0.1 |
|  | Holosporaceae | 0.2 | 0.2 | 0.1 | 0.1 | 0.2 | 0.4 |
|  | Geodermatophilaceae | 0.2 | 0.0 | 0.0 | 0.2 | 0.4 | 0.2 |
|  | Alcaligenaceae | 0.2 | 0.3 | 0.1 | 0.2 | 0.2 | 0.2 |
|  | Desulfurellaceae | 0.2 | 0.2 | 0.1 | 0.2 | 0.2 | 0.3 |
|  | Polyangiaceae | 0.2 | 0.2 | 0.3 | 0.3 | 0.1 | 0.0 |
|  | Cryomorphaceae | 0.2 | 0.3 | 0.0 | 0.1 | 0.4 | 0.0 |
|  | Staphylococcaceae | 0.2 | 0.0 | 0.0 | 0.1 | 0.5 | 0.0 |
|  | OPB56 | 0.2 | 0.0 | 0.0 | 0.1 | 0.1 | 0.6 |
|  | CHAB-XI-27 | 0.2 | 0.1 | 0.3 | 0.0 | 0.1 | 0.4 |
|  | Bacillaceae | 0.2 | 0.0 | 0.1 | 0.3 | 0.1 | 0.2 |
|  | PHOS-HE51 | 0.2 | 0.2 | 0.2 | 0.2 | 0.2 | 0.0 |
|  | Methylobacteriaceae | 0.2 | 0.2 | 0.1 | 0.1 | 0.4 | 0.0 |
|  | Bdellovibrionaceae | 0.2 | 0.0 | 0.1 | 0.2 | 0.1 | 0.2 |
|  | Streptomycetaceae | 0.2 | 0.0 | 0.0 | 0.4 | 0.2 | 0.0 |
|  | Neisseriaceae | 0.1 | 0.2 | 0.0 | 0.0 | 0.3 | 0.2 |
|  | Myxococcaceae | 0.1 | 0.2 | 0.3 | 0.0 | 0.0 | 0.4 |
|  | Microbacteriaceae | 0.1 | 0.1 | 0.1 | 0.3 | 0.1 | 0.0 |
|  | KD3-93 | 0.1 | 0.0 | 0.1 | 0.2 | 0.2 | 0.0 |
|  | Hyphomonadaceae | 0.1 | 0.4 | 0.1 | 0.0 | 0.2 | 0.0 |
|  | TJ1 | 0.1 | 0.2 | 0.1 | 0.2 | 0.0 | 0.1 |
|  | Rhodobacteraceae | 0.1 | 0.3 | 0.1 | 0.1 | 0.1 | 0.1 |
|  | Desulfovibrionaceae | 0.1 | 0.4 | 0.1 | 0.2 | 0.0 | 0.0 |
|  | Chromatiaceae | 0.1 | 0.2 | 0.0 | 0.1 | 0.1 | 0.4 |
|  | Kineosporiaceae | 0.1 | 0.0 | 0.1 | 0.1 | 0.3 | 0.0 |
|  | unclassified | 0.1 | 0.2 | 0.0 | 0.2 | 0.0 | 0.1 |
|  | Erythrobacteraceae | 0.1 | 0.1 | 0.2 | 0.0 | 0.2 | 0.0 |
|  | DA111 | 0.1 | 0.0 | 0.1 | 0.2 | 0.2 | 0.0 |
|  | Moraxellaceae | 0.1 | 0.5 | 0.0 | 0.0 | 0.2 | 0.0 |
|  | Rhizobiaceae | 0.1 | 0.3 | 0.1 | 0.2 | 0.0 | 0.0 |
|  | Parachlamydiaceae | 0.1 | 0.2 | 0.1 | 0.2 | 0.0 | 0.1 |
|  | Intrasporangiaceae | 0.1 | 0.1 | 0.3 | 0.1 | 0.1 | 0.0 |
|  | Halothiobacillaceae | 0.1 | 0.2 | 0.1 | 0.1 | 0.1 | 0.1 |
|  | mle1-27 | 0.1 | 0.0 | 0.1 | 0.1 | 0.2 | 0.1 |
|  | Coriobacteriales | 0.1 | 0.0 | 0.1 | 0.1 | 0.2 | 0.1 |
|  | Coriobacteriaceae | 0.1 | 0.0 | 0.1 | 0.1 | 0.2 | 0.1 |
|  | Ruminococcaceae | 0.1 | 0.2 | 0.3 | 0.0 | 0.0 | 0.0 |
|  | Desulfuromonadaceae | 0.1 | 0.0 | 0.4 | 0.0 | 0.0 | 0.1 |
|  | 0319-6G20 | 0.1 | 0.1 | 0.1 | 0.1 | 0.1 | 0.0 |
|  | Sporichthyaceae | 0.1 | 0.0 | 0.1 | 0.2 | 0.0 | 0.0 |
|  | Planococcaceae | 0.1 | 0.0 | 0.0 | 0.1 | 0.2 | 0.1 |
|  | Others (<20) | 3.8 | 7.1 | 4.8 | 2.0 | 5.0 | 2.3 |

**Table S2. Most abundant genera.**

|  | Total | A1 | A2 | O1 | O2 | O3 |
| --- | --- | --- | --- | --- | --- | --- |
| uncultured Acidobacteriaceae | 6.1 | 6.9 | 14.5 | 4.2 | 3.5 | 5.5 |
| uncultured Gemmatimonadaceae | 3.9 | 4.1 | 3.0 | 4.0 | 2.2 | 6.5 |
| uncultured Nitrosomonadaceae | 3.2 | 3.8 | 2.7 | 2.9 | 1.7 | 5.6 |
| unclassified KD4-96 (Chloroflexi) | 2.9 | 4.2 | 2.2 | 1.8 | 4.2 | 2.5 |
| uncultured Xanthobacteraceae | 2.8 | 1.8 | 2.1 | 5.4 | 2.6 | 0.3 |
| Candidatus_Solibacter | 2.8 | 1.8 | 3.9 | 2.8 | 2.6 | 2.5 |
| Flexibacter | 2.4 | 2.8 | 0.3 | 3.3 | 2.6 | 2.3 |
| Beggiatoa | 2.2 | 0.3 | 0.0 | 0.0 | 0.0 | 11.6 |
| unclassified OPB35 (Verrucomicrobia) | 1.9 | 2.1 | 1.0 | 0.9 | 4.5 | 0.4 |
| Gemmatimonas | 1.8 | 0.5 | 2.3 | 1.1 | 2.0 | 3.2 |
| Solirubrobacter | 1.8 | 0.1 | 0.4 | 2.3 | 3.7 | 0.5 |
| Geobacter | 1.4 | 2.1 | 7.6 | 0.0 | 0.0 | 0.0 |
| Bradyrhizobium | 1.3 | 0.1 | 0.1 | 4.0 | 0.2 | 0.0 |
| unclassified S085 (Chloroflexi) | 1.3 | 2.0 | 1.5 | 1.0 | 1.8 | 0.5 |
| Candidatus_Chloroacidobacterium | 1.3 | 2.1 | 0.7 | 0.4 | 1.4 | 2.3 |
| Sphingomonas | 1.2 | 0.8 | 1.2 | 1.0 | 1.4 | 1.7 |
| uncultured Chitinophagaceae | 1.2 | 0.3 | 1.6 | 1.5 | 1.4 | 0.6 |
| unclassified candidate division TM7 | 1.1 | 1.0 | 0.8 | 0.9 | 1.2 | 1.7 |
| Flavisolibacter | 1.1 | 0.2 | 0.4 | 0.1 | 3.4 | 0.5 |
| Haliangium | 1.0 | 0.7 | 1.4 | 0.6 | 1.7 | 0.6 |
| Thiobacillus | 1.0 | 0.1 | 0.1 | 0.0 | 0.3 | 4.6 |
| Sphingomonas | 0.9 | 0.6 | 0.7 | 0.8 | 1.2 | 1.3 |
| uncultured Methylocystaceae | 0.9 | 1.5 | 0.9 | 1.1 | 1.0 | 0.1 |
| unclassified uncultured GR-WP-33-30 (Deltaproteobacteria) | 0.9 | 1.3 | 1.5 | 1.0 | 0.6 | 0.2 |
| Nordella | 0.8 | 2.5 | 1.5 | 0.5 | 0.4 | 0.4 |
| unclassified 32-20 (Holophagae) | 0.8 | 1.2 | 2.0 | 0.9 | 0.4 | 0.0 |
| uncultured Sinobacteraceae | 0.8 | 1.6 | 0.7 | 1.1 | 0.4 | 0.3 |
| unclassified TK10 (Chloroflexi) | 0.7 | 0.9 | 0.2 | 0.3 | 0.4 | 2.1 |
| Aquicella | 0.7 | 1.3 | 0.6 | 0.4 | 0.5 | 1.0 |
| Steroidobacter | 0.7 | 0.5 | 0.4 | 0.5 | 0.3 | 1.8 |
| unclassified 196up (Gammaproteobacteria) | 0.7 | 0.1 | 0.1 | 0.1 | 0.0 | 3.2 |
| unclassified candidate division TM7 | 0.7 | 0.1 | 0.7 | 0.6 | 0.3 | 1.4 |
| unclassified DA101 (Spartobacteria) | 0.6 | 0.0 | 0.4 | 0.8 | 1.4 | 0.0 |
| Chitinophaga | 0.6 | 0.1 | 0.3 | 0.8 | 0.8 | 0.5 |
| Edaphobacter | 0.6 | 0.1 | 0.1 | 0.2 | 0.0 | 2.7 |
| Mycobacterium | 0.6 | 0.3 | 0.1 | 1.1 | 0.8 | 0.1 |
| unclassified wr0007 (Rhodospirillales) | 0.6 | 0.3 | 0.6 | 0.6 | 1.0 | 0.2 |
| Candidatus_Koribacter | 0.5 | 0.2 | 1.0 | 0.9 | 0.1 | 0.3 |
| unclassified SC-I-84 (Betaproteobacteria) | 0.5 | 0.6 | 1.1 | 0.4 | 0.4 | 0.5 |
| Nitrospira | 0.5 | 1.2 | 1.2 | 0.2 | 0.4 | 0.3 |
| unclassified iii1-8 (Holophagae) | 0.5 | 1.7 | 0.4 | 0.2 | 0.6 | 0.3 |
| unclassified TRA3-20 (Betaproteobacteria) | 0.5 | 0.6 | 0.1 | 0.5 | 0.7 | 0.5 |
| unclassified candidate division WS3 | 0.5 | 0.6 | 1.4 | 0.2 | 0.0 | 0.7 |
| unclassified WCHB1-25 (Lentisphaeria) | 0.4 | 0.1 | 0.2 | 0.2 | 0.0 | 1.6 |
| Flavobacterium | 0.4 | 1.2 | 1.0 | 0.4 | 0.1 | 0.1 |
| Acidothermus | 0.4 | 0.0 | 0.0 | 1.1 | 0.2 | 0.1 |
| Devosia | 0.4 | 0.3 | 0.3 | 0.7 | 0.2 | 0.1 |
| unclassified WCHB1-60 | 0.4 | 0.3 | 0.6 | 0.3 | 0.4 | 0.4 |
| unclassified Lineage IV (Candidate division TG1) | 0.4 | 0.2 | 0.3 | 0.6 | 0.4 | 0.2 |
| Rhodobium | 0.4 | 0.7 | 0.3 | 0.4 | 0.3 | 0.2 |
| Phenylobacterium | 0.3 | 0.4 | 0.3 | 0.4 | 0.5 | 0.1 |
| Terriglobus | 0.3 | 0.1 | 0.1 | 0.8 | 0.2 | 0.1 |
| uncultured Anaerolinaceae | 0.3 | 1.3 | 0.8 | 0.1 | 0.2 | 0.1 |
| Iamia | 0.3 | 0.3 | 0.1 | 0.3 | 0.6 | 0.2 |
| unclassified RB25 (Acidobacteria) | 0.3 | 0.3 | 1.1 | 0.1 | 0.4 | 0.1 |
| unclassified env.OPS_17 (Spartobacteria) | 0.3 | 0.4 | 0.3 | 0.3 | 0.4 | 0.1 |
| Niabella | 0.3 | 0.2 | 0.3 | 0.5 | 0.3 | 0.1 |
| unclassified candidate division OP10 | 0.3 | 0.4 | 0.1 | 0.0 | 0.6 | 0.5 |
| unclassified MND8 (Rhodospirillales) | 0.3 | 0.6 | 0.2 | 0.5 | 0.2 | 0.0 |
| Roseiflexus | 0.3 | 1.2 | 0.3 | 0.2 | 0.3 | 0.0 |
| unclassified JG37-AG-15 (Myxococcales) | 0.3 | 0.5 | 0.1 | 0.1 | 0.6 | 0.3 |
| unclassified GIF3 (Chloroflexi) | 0.3 | 0.4 | 0.7 | 0.1 | 0.4 | 0.0 |
| Lysobacter | 0.3 | 0.5 | 0.1 | 0.0 | 0.0 | 1.0 |
| Segetibacter | 0.3 | 0.2 | 0.0 | 0.2 | 0.8 | 0.0 |
| unclassified SJA-36 (Holophagae) | 0.3 | 0.1 | 0.6 | 0.5 | 0.1 | 0.0 |
| Acidobacterium | 0.3 | 0.0 | 0.2 | 0.7 | 0.1 | 0.0 |
| unclassified candidate division OD1 | 0.3 | 0.3 | 0.0 | 0.1 | 0.4 | 0.6 |
| Burkholderia | 0.3 | 0.0 | 0.0 | 0.5 | 0.3 | 0.1 |
| Methylosinus | 0.2 | 0.4 | 0.4 | 0.3 | 0.2 | 0.0 |
| Pseudonocardia | 0.2 | 0.1 | 0.7 | 0.2 | 0.3 | 0.1 |
| unclassified SJA-28 (Chlorobiales) | 0.2 | 0.3 | 0.1 | 0.1 | 0.6 | 0.0 |
| uncultured Fibrobacteraceae | 0.2 | 0.4 | 0.0 | 0.1 | 0.6 | 0.0 |
| Cytophaga | 0.2 | 0.1 | 0.0 | 0.7 | 0.1 | 0.0 |
| Anaeromyxobacter | 0.2 | 0.2 | 0.8 | 0.1 | 0.3 | 0.0 |
| Frankia | 0.2 | 0.1 | 0.1 | 0.3 | 0.5 | 0.0 |
| Sediminibacterium | 0.2 | 0.1 | 0.1 | 0.5 | 0.2 | 0.0 |
| Pseudomonas | 0.2 | 0.9 | 0.2 | 0.1 | 0.1 | 0.2 |
| uncultured Acetobacteraceae | 0.2 | 0.0 | 0.3 | 0.6 | 0.1 | 0.0 |
| Uliginosibacterium | 0.2 | 0.5 | 0.0 | 0.0 | 0.0 | 0.9 |
| uncultured Bradyrhizobiaceae | 0.2 | 0.1 | 0.1 | 0.6 | 0.1 | 0.0 |
| Legionella | 0.2 | 0.1 | 0.0 | 0.1 | 0.4 | 0.4 |
| Holospora | 0.2 | 0.2 | 0.1 | 0.1 | 0.2 | 0.4 |
| unclassified MNH4 (Rhodospirillales) | 0.2 | 0.3 | 0.4 | 0.2 | 0.1 | 0.1 |
| Candidatus_Magnetobacterium | 0.2 | 0.3 | 0.7 | 0.1 | 0.2 | 0.0 |
| Azonexus | 0.2 | 0.3 | 0.0 | 0.0 | 0.0 | 0.8 |
| Solitalea | 0.2 | 0.1 | 0.1 | 0.1 | 0.5 | 0.0 |
| Variovorax | 0.2 | 0.1 | 0.2 | 0.4 | 0.1 | 0.1 |
| Solimonas | 0.2 | 0.1 | 0.1 | 0.1 | 0.4 | 0.1 |
| uncultured Beijerinckiaceae | 0.2 | 0.1 | 0.1 | 0.6 | 0.0 | 0.0 |
| Phaselicystis | 0.2 | 0.3 | 0.4 | 0.2 | 0.1 | 0.0 |
| Labrys | 0.2 | 0.5 | 0.5 | 0.2 | 0.0 | 0.0 |
| Terrimonas | 0.2 | 0.1 | 0.1 | 0.1 | 0.4 | 0.2 |
| Chthoniobacter | 0.2 | 0.3 | 0.1 | 0.1 | 0.2 | 0.2 |
| unclassified AKYG1722 (Thermomicrobia) | 0.2 | 0.1 | 0.1 | 0.2 | 0.3 | 0.0 |
| Marmoricola | 0.2 | 0.1 | 0.1 | 0.2 | 0.4 | 0.0 |
| Hyphomicrobium | 0.2 | 0.5 | 0.1 | 0.3 | 0.1 | 0.0 |
| Denitratisoma | 0.2 | 0.4 | 0.2 | 0.1 | 0.0 | 0.3 |
| Candidatus_Odyssella | 0.2 | 0.4 | 0.2 | 0.0 | 0.1 | 0.3 |
| uncultured (Rhodospirillaceae) | 0.2 | 0.1 | 0.1 | 0.3 | 0.2 | 0.2 |
| unklassified NKB5 (Gammaproteobacteria) | 0.2 | 0.3 | 0.1 | 0.1 | 0.2 | 0.2 |
| unclassified TA06 | 0.2 | 0.0 | 0.1 | 0.5 | 0.0 | 0.0 |
| Staphylococcus | 0.2 | 0.0 | 0.0 | 0.1 | 0.5 | 0.0 |
| Clostridium | 0.2 | 0.4 | 0.8 | 0.0 | 0.0 | 0.0 |
| unclassified OPB56 | 0.2 | 0.0 | 0.0 | 0.1 | 0.1 | 0.6 |
| uncultured Alcaligenaceae | 0.2 | 0.2 | 0.1 | 0.2 | 0.2 | 0.1 |
| unclassified CHAB-XI-27 | 0.2 | 0.1 | 0.3 | 0.0 | 0.1 | 0.4 |
| unclassified PHOS-HE51 | 0.2 | 0.2 | 0.2 | 0.2 | 0.2 | 0.0 |
| Niastella | 0.2 | 0.1 | 0.7 | 0.1 | 0.1 | 0.0 |
| Pedomicrobium | 0.2 | 0.2 | 0.1 | 0.2 | 0.2 | 0.1 |
| Aspromonas | 0.2 | 0.0 | 0.1 | 0.0 | 0.0 | 0.6 |
| Singularimonas | 0.2 | 0.0 | 0.0 | 0.0 | 0.1 | 0.7 |
| Dokdonella | 0.2 | 0.3 | 0.1 | 0.3 | 0.1 | 0.1 |
| uncultured Caldilineaceae | 0.2 | 0.4 | 0.2 | 0.1 | 0.2 | 0.0 |
| Paenibacillus | 0.1 | 0.0 | 0.1 | 0.1 | 0.4 | 0.0 |
| Arenimonas | 0.1 | 0.1 | 0.5 | 0.1 | 0.1 | 0.0 |
| Nocardioides | 0.1 | 0.0 | 0.1 | 0.2 | 0.2 | 0.0 |
| Azospirillum | 0.1 | 0.0 | 0.1 | 0.1 | 0.2 | 0.2 |
| Massilia | 0.1 | 0.0 | 0.0 | 0.4 | 0.1 | 0.0 |
| Nitrosospira | 0.1 | 0.0 | 0.0 | 0.0 | 0.0 | 0.7 |
| Bdellovibrio | 0.1 | 0.0 | 0.1 | 0.2 | 0.1 | 0.2 |
| uncultured Xanthomonadaceae | 0.1 | 0.0 | 0.4 | 0.0 | 0.2 | 0.2 |
| Pyxidicoccus | 0.1 | 0.2 | 0.3 | 0.0 | 0.0 | 0.4 |
| Sphingosinicella | 0.1 | 0.0 | 0.0 | 0.3 | 0.2 | 0.0 |
| Hoeflea | 0.1 | 0.2 | 0.0 | 0.3 | 0.1 | 0.0 |
| Candidatus_Alysiosphaera | 0.1 | 0.2 | 0.1 | 0.1 | 0.2 | 0.1 |
| uncultured Desulfurellaceae | 0.1 | 0.1 | 0.0 | 0.2 | 0.1 | 0.3 |
| Mucilaginibacter | 0.1 | 0.0 | 0.0 | 0.3 | 0.1 | 0.0 |
| Mesorhizobium | 0.1 | 0.0 | 0.0 | 0.3 | 0.1 | 0.0 |
| Methylibium | 0.1 | 0.3 | 0.2 | 0.1 | 0.1 | 0.0 |
| unclassified KD3-93 | 0.1 | 0.0 | 0.1 | 0.2 | 0.2 | 0.0 |
| Arthrobacter | 0.1 | 0.0 | 0.0 | 0.3 | 0.1 | 0.0 |
| Rothia | 0.1 | 0.0 | 0.0 | 0.0 | 0.5 | 0.0 |
| Methylocystis | 0.1 | 0.1 | 0.2 | 0.0 | 0.3 | 0.0 |
| unclassified SHA26(Chloroflexi) | 0.1 | 0.0 | 0.0 | 0.1 | 0.3 | 0.0 |
| Streptomyces | 0.1 | 0.0 | 0.0 | 0.3 | 0.2 | 0.0 |
| uncultured Planctomycetaceae | 0.1 | 0.2 | 0.0 | 0.1 | 0.2 | 0.1 |
| Cohnella | 0.1 | 0.0 | 0.0 | 0.1 | 0.3 | 0.0 |
| Bilophila | 0.1 | 0.4 | 0.1 | 0.2 | 0.0 | 0.0 |
| unclassified TJ1 (Rhodospirillales) | 0.1 | 0.2 | 0.1 | 0.2 | 0.0 | 0.1 |
| Acidisphaera | 0.1 | 0.4 | 0.1 | 0.2 | 0.0 | 0.1 |
| Geothermobacter | 0.1 | 0.3 | 0.2 | 0.0 | 0.2 | 0.0 |
| uncultured Caulobacteraceae | 0.1 | 0.0 | 0.2 | 0.1 | 0.2 | 0.0 |
| Kineosporia | 0.1 | 0.0 | 0.1 | 0.1 | 0.3 | 0.0 |
| Flexithrix | 0.1 | 0.1 | 0.1 | 0.0 | 0.3 | 0.1 |
| unclassified SAR203 (Chloroflexi) | 0.1 | 0.2 | 0.0 | 0.2 | 0.0 | 0.1 |
| unclassified DA111 (Rhodospirillales) | 0.1 | 0.0 | 0.1 | 0.2 | 0.2 | 0.0 |
| Blastococcus | 0.1 | 0.0 | 0.0 | 0.1 | 0.2 | 0.1 |
| Oxalophagus | 0.1 | 0.0 | 0.0 | 0.2 | 0.1 | 0.1 |
| Ideonella | 0.1 | 0.2 | 0.1 | 0.2 | 0.0 | 0.0 |
| Bacillus | 0.1 | 0.0 | 0.0 | 0.2 | 0.0 | 0.2 |
| Hirschia | 0.1 | 0.3 | 0.1 | 0.0 | 0.2 | 0.0 |
| Afifella | 0.1 | 0.2 | 0.2 | 0.1 | 0.0 | 0.0 |
| Geopsychrobacter | 0.1 | 0.0 | 0.1 | 0.2 | 0.1 | 0.1 |
| unclassified TA06 | 0.1 | 0.9 | 0.0 | 0.0 | 0.0 | 0.0 |
| Fulvivirga | 0.1 | 0.1 | 0.0 | 0.2 | 0.0 | 0.1 |
| Sphingobium | 0.1 | 0.2 | 0.0 | 0.1 | 0.1 | 0.1 |
| unclassified mle1-27 (Myxococcales) | 0.1 | 0.0 | 0.1 | 0.1 | 0.2 | 0.1 |
| Sorangium | 0.1 | 0.0 | 0.1 | 0.3 | 0.0 | 0.0 |
| Microlunatus | 0.1 | 0.0 | 0.0 | 0.2 | 0.1 | 0.0 |
| Xanthobacter | 0.1 | 0.0 | 0.0 | 0.2 | 0.1 | 0.0 |
| Owenweeksia | 0.1 | 0.1 | 0.0 | 0.1 | 0.2 | 0.0 |
| unclassified O319-6G20 (Myxococcales) | 0.1 | 0.1 | 0.1 | 0.1 | 0.1 | 0.0 |
| Sporichthya | 0.1 | 0.0 | 0.1 | 0.2 | 0.0 | 0.0 |
| Leifsonia | 0.1 | 0.0 | 0.1 | 0.2 | 0.1 | 0.0 |
| Luedemannella | 0.1 | 0.0 | 0.0 | 0.2 | 0.1 | 0.0 |
| Thiovirga | 0.1 | 0.2 | 0.1 | 0.1 | 0.1 | 0.0 |
| uncultured Methylobacteriaceae | 0.1 | 0.1 | 0.1 | 0.0 | 0.2 | 0.0 |

Table S3. Spearman's correlation coefficients between plant and bacterial diversity.

|  | Plant sp. observed |
| --- | --- |
| Bact. sp. observed 0.03 | r=-0.2052, p=0.74 |
| Bact. Chao1 0.03 | r=-0.0523, p=0.93 |
| Bact. ACE 0.03 | r=-0.0513, p=0.93 |
| Bact. Shannon H' 0.03 | r=-0.2052, p=0.74 |
| Bact. sp. observed 0.10 | r=-0.2052, p=0.74 |
| Bact. Chao1 0.10 | r=-0.2052, p=0.74 |
| Bact. ACE 0.10 | r=0.3591, p=0.55 |
| Bact. Shannon H' 0.10 | r=-0.3421, p=0.57 |
| Bact. phylodiversity | r=-0.2052, p=0.74 |

Figure S1
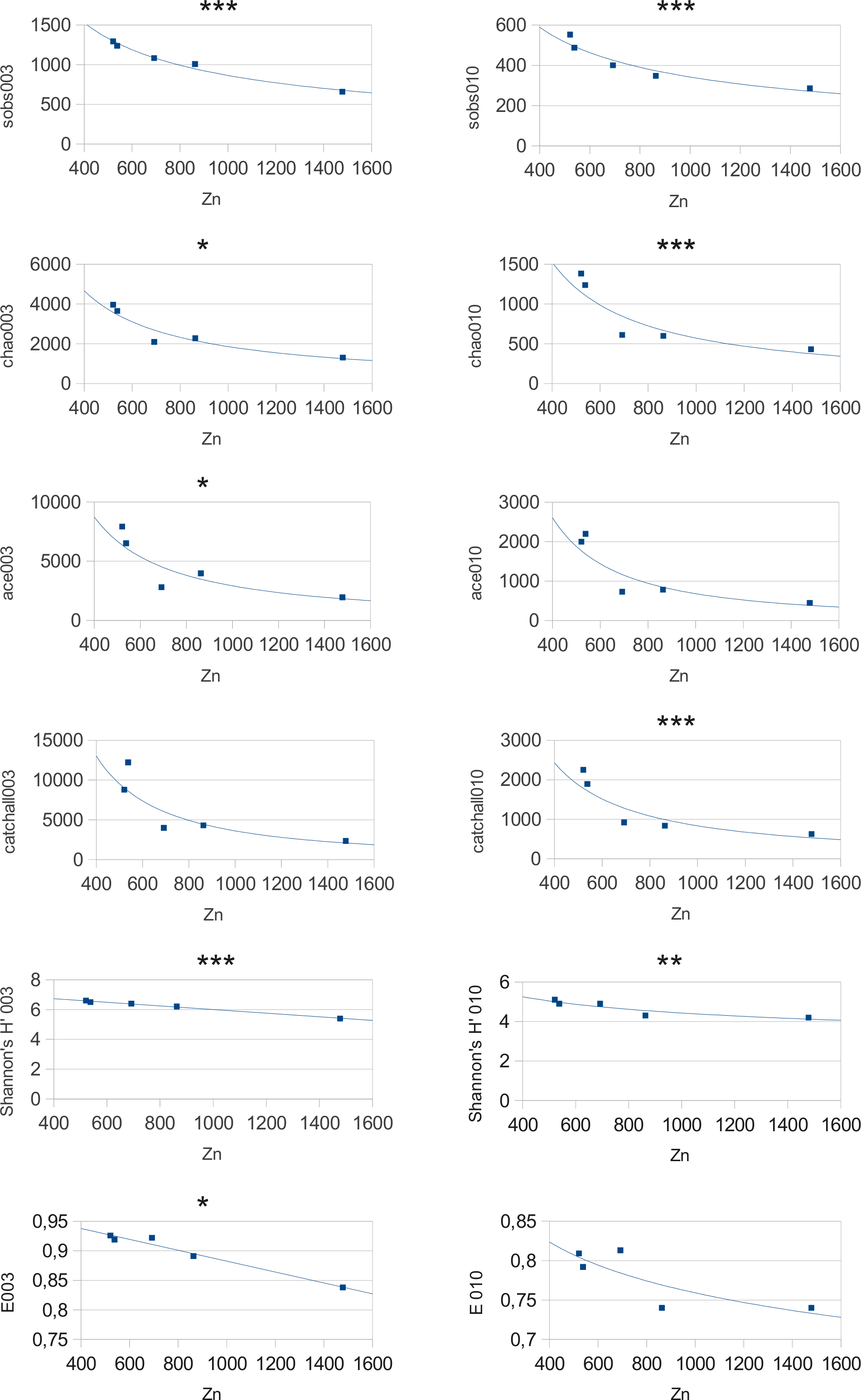
. Correlation of bacterial species richness, diversity and evennes with bioavailable Zn concentration. * p<0.05, ** p<0.01, *** p<0.001.

Figure S2. Collectors curves for Chao1 and ACE total species richness estimators.


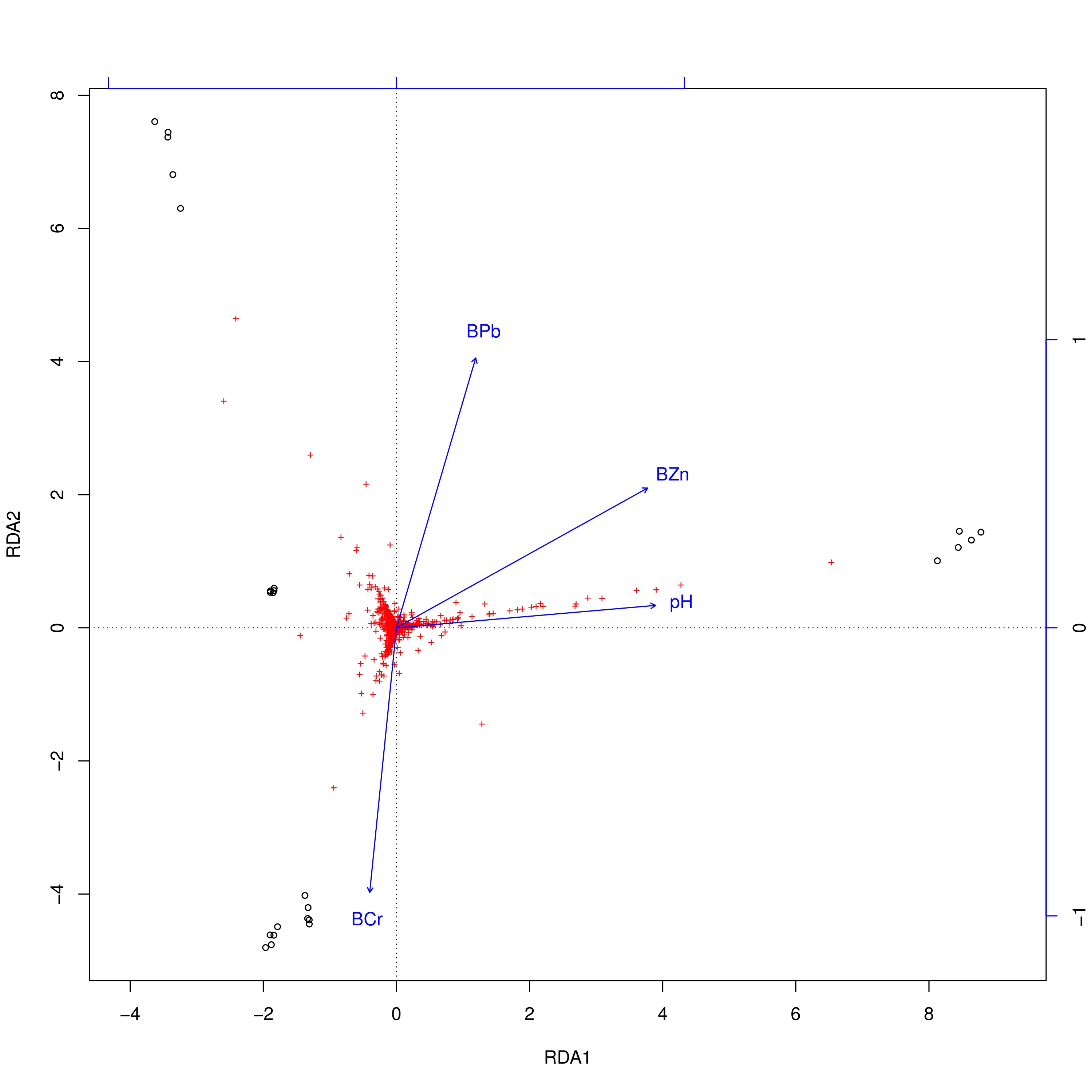

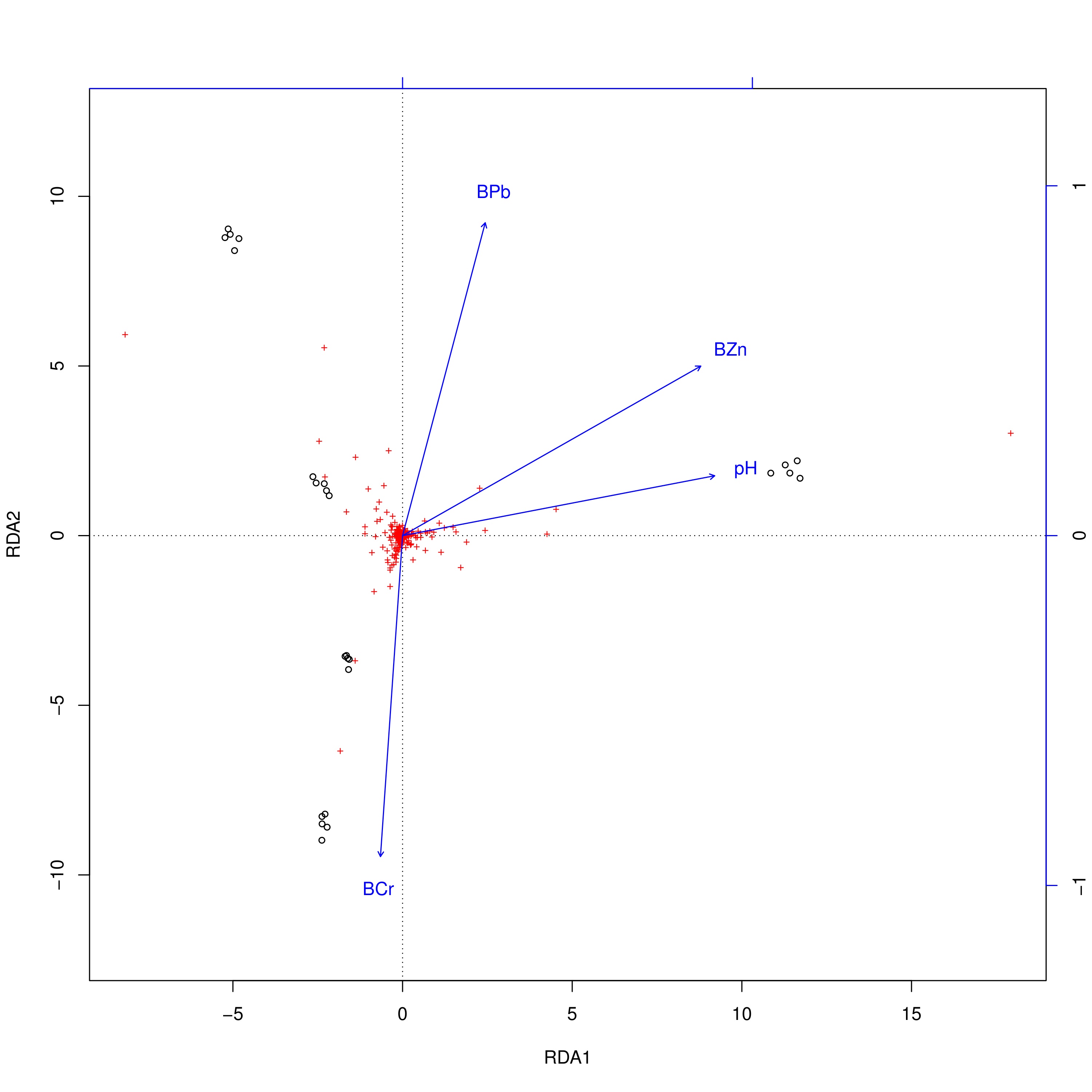


Figure S3. Biplots of RDA of shared OTU matrices constructed at the 0.03 (left) and 0.10 (right) clustering levels. Only significant environmental variables are shown.
